# *Wolbachia pipientis* grows in *Saccharomyces cerevisiae* evoking early death of the host and deregulation of mitochondrial metabolism

**DOI:** 10.1002/mbo3.675

**Published:** 2018-06-13

**Authors:** Cristina Uribe‐Alvarez, Natalia Chiquete‐Félix, Lilia Morales‐García, Arlette Bohórquez‐Hernández, Norma Laura Delgado‐Buenrostro, Luis Vaca, Antonio Peña, Salvador Uribe‐Carvajal

**Affiliations:** ^1^ Depto. de Genética Molecular Instituto de Fisiología Celular Universidad Nacional Autónoma de México Ciudad de México México; ^2^ Depto. de Biología Celular y del Desarrollo Instituto de Fisiología Celular Universidad Nacional Autónoma de México Ciudad de México México; ^3^ Unidad de Biomedicina UBIMED Facultad de Estudios Superiores Iztacala Universidad Nacional Autónoma de México Tlanepantla Edo. de México México

**Keywords:** bioenergetics, endosymbiosis, oxidative phosphorylation, *Saccharomyces cerevisiae*, *Wolbachia pipientis*

## Abstract

*Wolbachia* sp. has colonized over 70% of insect species, successfully manipulating host fertility, protein expression, lifespan, and metabolism. Understanding and engineering the biochemistry and physiology of *Wolbachia* holds great promise for insect vector‐borne disease eradication. *Wolbachia* is cultured in cell lines, which have long duplication times and are difficult to manipulate and study. The yeast strain *Saccharomyces cerevisiae* W303 was used successfully as an artificial host for *Wolbachia* *w*AlbB. As compared to controls, infected yeast lost viability early, probably as a result of an abnormally high mitochondrial oxidative phosphorylation activity observed at late stages of growth. No respiratory chain proteins from *Wolbachia* were detected, while several *Wolbachia* F_1_F_0_‐ATPase subunits were revealed. After 5 days outside the cell, *Wolbachia* remained fully infective against insect cells.

## 1 INTRODUCTION

Construction of artificial ecosystems mimicking symbiotic relationships have been proposed to study ecology and evolution of symbioses (Hosoda et al., 2011; Momeni, Chen, Hillesland, Waite, & Shou, 2011), to engineer microbial consortia (Brenner, You, & Arnold, 2008; French, 2017; Frey‐Klett et al., 2011; Mee & Wang, 2012), and to host uncultivable bacteria (Stewart, 2012). Synthetic mutualism of species that do not interact naturally has been established in coculture between bacteria, yeast, amoeba, alga, cell lines, and tissues (Buchsbaum & Buchsbaum, 1934; Hosoda & Yomo, 2011; Hosoda et al., 2011; Kubo et al., 2013; Lőrincz et al., 2010; Shou, Ram, & Vilar, 2007). Several bacterial endosymbionts have been found in yeast (Kang, Jeon, Hwang, & Park, 2009; Saniee & Siavoshi, 2015) as well as in fungal hyphae and spores (Bertaux et al., 2003; Bianciotto et al., 2004; de Boer et al., 2004; Hoffman & Arnold, 2010; Lumini, Ghignone, Bianciotto, & Bonfante, 2006; Partida‐Martinez & Hertweck, 2005; Sato et al., 2010). In this work, we cultured the obligate endosymbiont bacterium *Wolbachia* in an artificial host: the nonpathogenic yeast *Saccharomyces cerevisiae*.

*Wolbachia pipientis* is an exceedingly successful obligate endoparasite/endosymbiont in nematodes and arthropods (Taylor & Hoerauf, 1999; Werren, 1997; Werren, Baldo, & Clark, 2008). The size of the *Wolbachia* genome varies widely depending on the strain. Arthropod endoparasites have much larger genomes than nematode endosymbionts (Bandi, Slatko, & O'Neill, 1999; Darby et al., 2012; Foster et al., 2005; Klasson et al., 2008; Salzberg, Puiu, Sommer, Nene, & Lee, 2009; Wu et al., 2004). In regard to a possible aerobic metabolism, the *Wolbachia* from the plant hopper *Leodelphax stratellus* (*wStr*) is ten times more sensitive to paraquat than the insect host cell, suggesting that *wStr* does not possess the enzymes needed for reactive oxygen species (ROS) detoxification and thus it may be anaerobic or microaerophilic (Fallon, Kurtz, & Carroll, 2013). In contrast, eliminating *Wolbachia* with tetracycline in filaria, increases respiratory‐chain gene expression in the host and causes an early death. This result, lead to the hypothesis that at least in filariae *Wolbachia* contributes as an energy generator for the host (Strübing, Lucius, Hoerauf, & Pfarr, 2010; Darby et al., 2012, 2014).

Culturing obligate intracellular bacteria is a challenge. Insect cells support *Wolbachia* growth, but culture times are long and cells are difficult to manipulate. Alternative systems such as mammalian blood have proven helpful to grow intracellular organisms such as *Sodalis* (Dale & Maudlin, 1999). However, *Wolbachia* did not seem to grow in blood and this was not pursued further (Result not‐shown; see Methods). In contrast, *Saccharomyces cerevisiae* did support the growth of *Wolbachia* strain *w*AlbB.

As it can be extensively manipulated, *S. cerevisiae* is widely used as a model organism in biochemistry and molecular biology. In *S. cerevisiae*, it is possible to study processes such as the Crabtree effect observed in tumor cells (Diaz‐Ruiz, Rigoulet, & Devin, 2011) and to model cell death in response to anoxia or ischemia/reperfusion (Stella, Burgos, Chapela, & Gamondi, 2011). In addition, it is used as a host to study DNA and RNA viral replication (Alves‐Rodrigues, Galão, Meyerhans, & Díez, 1988), to identify and characterize bacterial effectors and toxins (Siggers & Lesser, 2008) and to analyze the function of heterologously expressed proteins such as the *Yarrowia lipolytica* and the mammalian brown‐fat mitochondrial uncoupling proteins (UCPs) (Guerrero‐Castillo et al., 2011). Thus, when it was observed that *Wolbachia* grew in *S. cerevisiae*, the system was characterized and the effects of *Wolbachia* infection on its host were analyzed.

Growing *Wolbachia* in insect cell cultures or in live hosts presents difficulties that have precluded detailed biochemistry and physiology studies (Baldridge et al., 2014; Khoo, Venard, Fu, Mercer, & Dobson, 2013). Here, we used the *S. cerevisiae* strain W303 as an alternative host for *Wolbachia w*AlbB and analyzed the host/endosymbiont system. Infected yeasts died earlier than controls. This probably resulted from an abnormally high mitochondrial oxidative phosphorylation activity observed at late stages of growth. Understanding *Wolbachia* and host‐*Wolbachia* interactions holds great promise for medical, parasitological, and biotechnological applications.

## 2 EXPERIMENTAL PROCEDURES

### 2.1 Aa23 cell line maintenance

Aa23 cell line (*Aedes albopictus* infected with *w*AlbB) (O'Neill et al., 1997) was kindly donated by Professor Anne Fallon (U. Minnesota) and maintained in Eagle's minimal essential medium (MEM, Sigma Chemical Co. M0643). MEM was supplemented as indicated elsewhere (Shih, Gerenday, & Fallon, 1998). The medium was filter‐sterilized (Millipore, 0.22 μm) and stored in 200 ml aliquots at 4°C. Prior to use, heat‐inactivated fetal bovine serum (FBS; 30 min at 56°C) was added to a final concentration of 10% (Shih et al., 1998). The insect cell line was grown on True Line TR 4003 140 mm sterile petri dishes at 27°C in a 5% CO_2_ atmosphere (ESCO CelCulture CO_2_ incubator or in Corning culture flasks, Shanghai, China). Subcultures were performed in a 1:10 split at 90% confluence. A sample from this cell line was treated with tetracycline to eliminate *Wolbachia* infection (Aa23Tet) (Dobson, Marsland, Veneti, Bourtzis, & O'Neill, 2002).

### 2.2 Cell viability assays

Viability of Aa23 cell line, *Wolbachia* or yeast was assessed using the BacLight live‐dead staining kit (Molecular Probes, Carlsbad, CA). Ten microliters of cell suspension were stained according to the manufacturer suggested protocol and viewed in an epifluorescence NIKON microscope.

### 2.3 Failed attempts to grow *Wolbachia* ex‐vivo and a serendipitous finding

The original idea was to find a system where *Wolbachia* would grow ex‐vivo. To do this, diverse protocols used for other endosymbionts such as *Sodalis* and *Coxiella* were followed (Dale & Maudlin, 1999; Omsland et al., 2009, 2013). It was found that some components did improve survival in isolated *Wolbachia*, even if we never observed substantial growth. Some of these agents were: (1) Trehalose and other compatible solutes such as mannitol, glycerol and sucrose, known to stabilize pollen (Crowe, Reid, & Crowe, 1996; Leslie, Israeli, Lighthart, Crowe, & Crowe, 1995) and isolated proteins (Sampedro & Uribe, 2004) (2) Actin, which supports binding and movements of some endosymbionts in vivo. (3) Catalase which deactivates hydrogen peroxide (Dale & Maudlin, 1999) and (4) Blood from large mammals, which has been used to grow *Sodalis* (Dale & Maudlin, 1999) and increases *Wolbachia* titers (Amuzu, Simmons, & McGraw, 2015; McMeniman, Hughes, & O'Neill, 2011). Human blood was also effective.

First, we tried growing *Wolbachia* using sheep blood. However, it was easily contaminated at the sites of extraction, so cultures had to be discarded often. On one occasion we obtained positive *wsp* gene amplification from a yeast colony grown in one of the agar plates. Out of curiosity, we studied the host, which turned out to be *S. cerevisiae*. From this accidental finding we decided to test a known strain of *S. cerevisiae* as an alternative host. We learned that, in order to support growth of *Wolbachia*, yeast culture media needed to be supplemented with blood, which eventually was substituted with ammonium ferric citrate with excellent results and none of the contamination problems. Neither compatible solutes, nor catalase nor actin enhanced growth. The second addition needed was bovine fetal serum, which was present in all original growth media but not in yeast culture media. FBS was titrated and we ended up using 1%.

**Figure 1:**
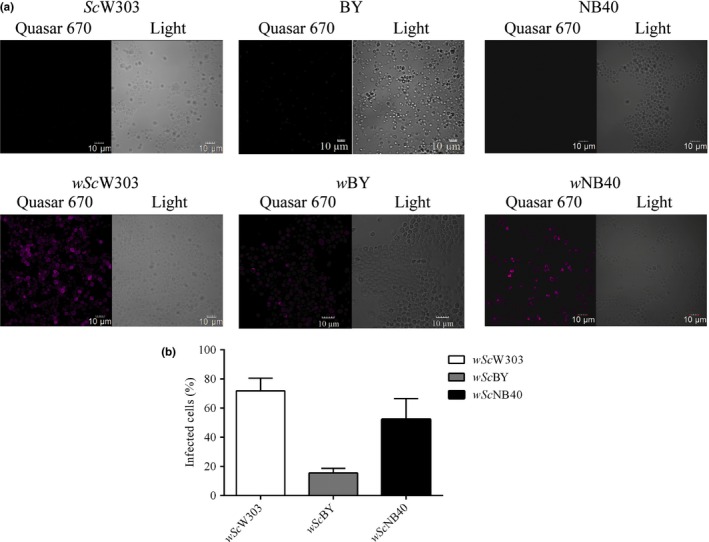
Infection of *Saccharomyces cerevisiae* with *Wolbachia*. (a) FISH using a *Wolbachia* 16S rDNA specific probe labeled with Quasar 670 (pink) was performed on 14 day old cultures of infected and control *S. cerevisiae strains* W303 (*Sc*W303), BY (*Sc*BY), and NB40 (*Sc*NB40) (b) After 14 days postinfection, the percentage of infected cells were counted as those with positive hybridization.

Among laboratory strains, infection was successful in W303 and NB40, while infection % in BY was milder (Figure 1). The *S. cerevisiae* strain W303‐1A (MATα; ura3‐1; trp1Δ 2; leu2‐3,112; his3‐11,15; ade2‐1; can1‐100) (Gutierrez‐Aguilar et al., 2014), where *Wolbachia* was abundant at 10 days of infection was chosen for further experiments (See Results).

### 2.4 *Wolbachia w*AlbB infection of the *Saccharomyces cerevisiae* W303 yeast strain (*wScW303*)

A first yeast infection was performed following a modified cell line infection protocol (Dobson et al., 2002). All procedures were performed under sterile conditions. The Aa23 cell line (containing *Wolbachia*) was grown in Corning cell culture flasks (225 cm^2^) as described in (Shih et al., 1998). After 20 days of culture, cells were scrapped and concentrated by centrifugation at 3,000*g* for 5 min. For homogenization, ~1*10^7^ cells were resuspended in 10 ml Eagles medium and vortexed for 10 min with (50% v/v) 3 mm sterile borosilicate glass beads (Rasgon, Gamston, & Ren, 2006). The homogenate was centrifuged at 3,000*g* for 10 min to remove unbroken cells. The supernatant was passed through a 2.7 μm syringe filter and the filtrate containing bacteria was centrifuged at 16,500*g* for 10 min. The pellet was resuspended in 2 ml of Mitsuhashi‐Maramorosch medium (MM) supplemented with 1 mmol L^−1^ ammonium ferric citrate and 20% fetal bovine serum (FBS) (MM Fe FBS). In parallel, yeasts were grown in a liquid YPD culture for 3 hr, harvested and centrifuged at 3,000*g* for 3 min. Culturing yeast in low oxygen environments prevents thickening of the cell wall (Aguilar‐Uscanga & Francois, 2000; Smith et al., 2000; Avrahami‐Moyal, Braun, & Engelberg, 2012). To induce contact between bacteria and yeast both bacteria (the whole 2 ml sample) and yeast (60 mg ww) were mixed and centrifuged at 2,500*g* for 1 hr at 20°C (Dobson et al., 2002). Bacteria‐infected yeast were plated (all 2 ml) on a Petri dish containing MM supplemented with 1 mmol L^−1^ ammonium ferric citrate plus 25% v/v outdated human packaged erythrocytes and 2% agar (MM Fe‐blood) and incubated at 27°C in a 5% CO_2_ chamber (ESCO, Cell Culture CO_2_ incubator, Singapore) for 14 days (Dale & Maudlin, 1999). Infection was confirmed by FISH and PCR. Infected yeast was transferred to a fresh agar plate every month for up to 6 months, then yeast was discarded and a new sample was used. Some aliquots were added with 40% glycerol, frozen and stored at −80°C, these samples have remained infective for nearly 10 months.

To transfer *Wolbachia* from yeast to yeast, slight modifications to the protocol were made: An aliquot of 100 μl of yeast taken from a glycerol‐frozen sample or a loophole of infected yeast cells was diluted in 2 ml YPD Fe 20% FBS and plated in YPD Fe‐blood agar plates, which were grown in 5% CO_2_. After 14 days, all cells grown in a Petri dish were collected and washed by centrifugation at 3,000*g* for 3 min at 20°C with sterile water and the pellet was suspended in 10 ml MM. The suspension was vortexed for 10 min in the presence of 0.425–0.600 mm sterile borosilicate glass beads (60% v/v) to disrupt yeast cells (note that beads were smaller than those used for insect cell lines). Disrupted yeasts were centrifuged at 3,000*g* for 10 min and the supernatant was centrifuged again 3,000*g* for 10 min. The washed supernatant was filtered through different 0.8–0.65–0.45 μm syringe filters. Again, we used filters with smaller pores than those used for cell lines due to the small size of yeast cells. The last filtrate was centrifuged at 16,500*g* for 10 min. The pellet (~60 mg ww) was suspended in 2 ml MM Fe FBS and used to infect yeast from 3‐h cultures as described above. The yeast–bacterium mixture was plated in a YPD Fe agar plate and incubated at 27°C with 5% CO_2_ for at least 7 days. Infection was evaluated using FISH and PCR.

### 2.5 Culture and maintenance of *w*AlbB*‐*infected *Saccharomyces cerevisiae* W303

Infected *S. cerevisiae* strains were kept in YPD plus 1 mmol L^−1^ ammonium ferric citrate agar plates. When transferring to liquid medium, a loophole from the desired strain was suspended in 100 ml of sterile YPDS and incubated at 28°C, 130 rpm for 48 hr. Precultures were decanted in one liter YPDS and incubated at the same conditions for up to 14 days. When transferring from solid to solid media, a loophole of yeast was suspended in 1 ml YPD supplemented with 1 mmol L^−1^ ammonium ferric citrate plus 20% FBS and plated on YPD agar. A cell passage every 2–3 weeks was performed in order to maintain the infection. When it was desired to eliminate *Wolbachia* from yeast, tetracycline 30 μg/ml was added five consecutive times to the medium as passages were performed (Dobson et al., 2002).

### 2.6 *Wolbachia w*AlbB infection of the C6C36 *Aedes albopictus* cell line

To determine whether *Wolbachia* cells retained its infective ability after all treatments, *Wolbachia* were isolated from *S. cerevisiae* grown in liquid YPD Fe 1% FBS and they were tested for infection against a C6C36 insect cell line.

### 2.7 *Wolbachia* surface protein (*wsp)* gene PCR identification

The *Wolbachia wsp* gene was amplified using the following primers: *wsp* 81F (5′ TGGTCCAATAAGTGATGAAGAAAC 3′) and *wsp* 691R (5′ AAAAATTAAACGCTACTCCA 3′) (Braig, Zhou, Dobson, & O'Neill, 1998) in a 25 μl reaction volume using recombinant Taq DNA polymerase (Thermo Fisher Scientific). PCR amplification was performed as reported elsewhere (Braig et al., 1998; Xi, Khoo, & Dobson, 2006). The PCR product was electrophoresed on a 1% agarose gel and stained with ethidium bromide. PCR product was purified using a GeneJET PCR purification Kit (Thermo Fisher Scientific) and sequenced in the Molecular Biology Unit at the Institute of Cellular Physiology, UNAM.

### 2.8 Fluorescence in‐situ hybridization (FISH)

*Wolbachia* 16S rDNA oligonucleotide probe labeled with Quasar 670 dye (λ_em_647, λ_ex_670) W1, 5′‐AATCCGGCCGARCCGACCC‐3′ was used for FISH assays (Heddi, Grenier, Khatchadourian, Charles, & Nardon, 1999). One milliliter of the desired culture was centrifuged at 3,000*g* for Aa23, C6C36 and yeast, and 18,500*g* for purified *Wolbachia* for 5 min. Protocol was followed as reported elsewhere (Genty, Bouchon, Raimond, & Bertaux, 2014). Samples were viewed in a FluoView FV‐1,000 Olympus confocal microscope, NA 1.4 with a 100X objective. Images were analyzed with the FV‐Viewer Olympus software.

### 2.9 Z‐cut images for cell reconstruction

Fourteen day old infected and noninfected yeast samples were visualized with a Olympus‐FV1000 or FV‐3000 microscopes. Z‐cut images were reconstructed using Imaris 7.2.1 and Image J software. Calcofluor‐white (0.05 mmol L^−1^ in 20% DMSO‐20 mmol L^−1^ Bicine Buffer) was used to stain fungus cell wall.

### 2.10 Antibodies

Primary antibodies: Mouse monoclonal Anti‐*Wolbachia* Surface Protein NR‐31029 was from BEI Resources, NIAID, NIH. Mouse monoclonal Anti‐VDAC was from Abcam. Secondary antibody: HRP coupled Anti‐mouse antibody from Jackson ImmunoResearch (West Grove, PA).

### 2.11 Western blot

A loophole from yeast grown in solid media was suspended in 200 μl of water; otherwise, 200 μl from liquid culture samples were centrifuged at 3,000*g* for 5 min and washed twice in water. The pellet was solubilized in 200 μl RIPA buffer (25 mmol L^−1^ Tris•HCl pH 7.6, 150 mmol L^−1^ NaCl, 1% NP‐40, 1% sodium deoxycholate, 0.1% SDS) supplemented with protease inhibitors 1 mmol L^−1^ PMSF (Sigma‐Aldrich) and Complete protease inhibitor cocktail (Roche‐CO‐RO) as recommended by the abcam protocol. Samples were lysed in a Sonics VibraCell sonicator (Sonics & materials, Inc., Newtown, CT) at 80% amplitude for 10 s and left under agitation in a Multi‐Vortex V‐32 (Biosan, Riga, Latvia) for 30 min at 4°C. Samples were centrifuged at 15,160*g* for 5 min. The supernatant was recovered and protein concentration was measured by Bradford in a PolarStar Omega (BMG labtech, Ortenberg, Germany) [(Bradford, 1976) #64]. Samples were diluted in a 4X buffer (500 mmol L^−1^ Tris, pH 6.8, 10% glycerol, 10% SDS, 0.05% beta‐mercapto‐ethanol, and 0.01% bromophenol blue) and boiled for 5 min. SDS/PAGE was performed in 10% polyacrylamide gels and electrotransferred to poly(vinylidenedifluoride) membranes as reported elsewhere (Chiquete‐Felix et al., 2009). Membranes were blocked with 5% Blotto nonfat dry milk in TBS‐T (50 mmol L^−1^ Tris, 104 mmol L^−1^ NaCl, pH 7.6, 0.1% Tween 20) for 1 hr, and incubated overnight at 4°C with the primary antibody. Membranes were washed with TBS‐T and incubated at 37°C for 1 hr with secondary antibody. Membranes were washed again and the bands were developed by chemiluminescence using an ECL kit (Amersham Biosciences, GE, Healthcare) (Chiquete‐Felix et al., 2009). PVDF membranes were stripped as indicated by abcam protocol using a mild‐stripping buffer, blocked with 5% Blotto nonfat dry milk in TBS‐T and reprobed with a different antibody as indicated.

### 2.12 Transmission electron microscopy of *wSc*W303

Infection was assessed by transmission electron microscopy (TEM) following a protocol from (Sun et al., 2015). Briefly, 500 μl of cells were harvested from 100 ml cultures of infected and uninfected *Saccharomyces cerevisiae* cultures form the first unintentional infection (*wSc*) at 10 days and *wSc*W303 of fourteen days. Yeast and *Wolbachia* samples were washed twice in distilled water at 740 g for 5 min for yeast and 23400 g for 10 min for bacteria in an Eppendorff Centrifuge 5415C. Samples were fixed in 2% KMnO_4_ at 4°C overnight. Next day, samples were washed for 15 min with deionized water six times and dehydrated with sequential 10‐minute washes with 50%, 70%, 80%, 90% ethanol and three washes with 100% ethanol. Samples were washed with ethanol‐propanone (1: 1) for 8 min, then with anhydrous propanone for 5 min, then with propanone‐EPON 821 (3: 1) for 1 hr and left in propanone‐EPON 821 (1 : 3) overnight. Next day, samples were concentrated and resuspended in propanone‐EPON 821 (1: 1) for 1 hr. Then, samples were concentrated again and left in resin for 24 hr. Then they were incubated for 12 hr at 37°C and then further incubated for 36 hr at 60°C. Resins were cut into 70 nm slices on an ultra‐microtome (Ultracut Reicheit‐jung) and observed in a JEOL JEM‐1200 EXII electron microscope. Data were processed using Gatan Digital Micrograph Software.

### 2.13 Mitochondrial (or Mitochondria/*Wolbachia* mixture) isolation

Yeast were centrifuged at 3,000*g* for 5 min, washed twice in water and resuspended in MES‐mannitol buffer (5 mmol L^−1^ MES, 0.6 mol L^−1^ mannitol, 0.1% BSA pH 6.8 adjusted with triethanolamine). Yeast were disrupted using a Bead Beater cell homogenizer (Biospec Products, OK, USA, final volume 50 ml) with 0.425–0.6 mm glass beads during three 20 s pulses separated by 40 s resting periods in ice (Uribe, Rangel, Espínola, & Aguirre, 1990). The homogenate was differentially centrifuged to isolate mitochondria similar to described in (Peña, Piña, Escamilla, & Piña, 1977). Briefly, cells were centrifuged at 1,100*g* for 5 min. The supernatant was centrifuged at 9,798*g* for 10 min and the pellet was resuspended in MES‐mannitol buffer and centrifuged at 3,000*g* for 5 min. Finally, the supernatant was centrifuged at 17,500*g* for 10 min. The resulting pellet was resuspended in minimal volume and protein concentration was measured by Biuret (Gornal, 1957) using a Beckman Coulter spectrophotometer at 540 nm.

### 2.14 Oxymetry

Mitochondrial high resolution respirometry was assessed in an Oroboros oxygraph (Oroboros Intrs Corp, Innsbruck, Austria) using 5 mmol L^−1^ MES, 0.6 mol L^−1^ mannitol pH 6.8, 10 mmol L^−1^ KCl and 4 mmol L^−1^ Pi at 30°C. Final volume in the closed chamber was 1.5 ml with a protein concentration of 0.5 mg prot/ml. Bacterial protein concentration of 0.5 mg prot/ml was used. The trace was started by the addition of 5 mmol L^−1^ of the indicated substrate: glycerol‐3‐phosphate, ethanol, NADH, pyruvate‐malate, succinate, glutamine or glutamate. For Complex IV evaluation, 5 mmol L^−1^ ascorbate (pH 7.6)‐ 0.05 mmol L^−1^ TMPD was used (Uribe, Ramirez, & Peña, 1985). Respiratory control was measured using 0.5 μl/ml ethanol to induce state II respiration and 1 mmol L^−1^ ADP to induce the phosphorylated state (Uribe et al., 1985). Respiratory chain inhibitors were used in the following concentrations: 0.1 μmol L^−1^ rotenone, 0.15 mmol L^−1^ flavone, 0.1 μmol L^−1^ antimycin A, and 2 mmol L^−1^ cyanide (Uribe et al., 1985).0.5 μmol L^−1^ CCCP was added as an uncoupler. Data were analyzed using the Oroboros Lab software.

### 2.15 Electrophoretic techniques and in‐gel activities

Blue native gel electrophoresis (BN‐PAGE) and high‐resolution clear native electrophoresis (hrCN‐PAGE) were performed as in (Wittig, Braun, & Schägger,^2006^; Wittig, Karas, & Schägger, 2007). Whole cells were solubilized with 2 mg dodecylmaltoside/mg protein plus 1 mmol L^−1^ PMSF and Complete protease inhibitor cocktail (Roche‐CO‐RO) and shaken for 30 min at 4°C. Membranes were centrifuged at 23,680*g* at 4°C for 1 hr. Protein concentration in the supernatants was determined by Bradford (1976). Between 0.1 and 0.15 mg of protein were loaded in 5%–15% polyacrylamide gradient gels. When hr‐CN PAGE electrophoresis was performed 0.01% Lauryl maltoside and 0.05% sodium deoxycholate were added to the cathode buffer [(Wittig et al., 2007) #69]. Gels were run for about an hour at 15 mA/gel in a Bio‐rad electrophoresis chamber. In‐gel NADH‐NBT oxido‐reductase (100 μg protein), succinate‐NBT oxido‐reductase (150 μg protein), cytochrome *c* oxidase (100 μg protein), and in‐gel ATPase (100 μg protein) activities were done as reported previously (Uribe‐Alvarez et al., 2016). 20 μg protein of solubilized Bovine Heart Mitochondria (BHM) were loaded in each gel as controls.

### 2.16 LC‐MALDI‐MS/MS

Indicated bands from hr‐CN PAGE or BN‐PAGE were enzymatically digested, separated on a HPLC EkspertnanoLC 425 (Eksigent, Redwood City CA) and analyzed in a MALDI‐TOF/TOF 4800 Plus mass spectrometer (ABSciex, Framingham MA) (Shevchenko, Tomas, Havli, Olsen, & Mann, 2006) in the Unidad de Genómica, Proteómica y Metabolómica, CINVESTAV‐IPN. Generated MS/MS spectra were compared using Protein Pilot software v. 4.0 (ABSciex, Framingham MA) against the *Saccharomyces cerevisiae* ATCC 204508 database (downloaded of Uniprot, 6721 protein sequences) and *Wolbachia* genus database (downloaded of Uniprot, 47781 protein sequences) using Paragon algorithm.

## 3 RESULTS

### 3.1 At the expense of its own viability, the artificial host *Saccharomyces cerevisiae* W303 supports growth of *Wolbachia w*AlbB

To study *Wolbachia* (*w*AlbB) large biomass yields plus a host that is easy to manipulate are needed. After testing different alternatives (see Methods), it was discovered that different *S. cerevisiae* strains were susceptible to infection and supported active *Wolbachia* proliferation. At 14 days of infection, *Wolbachia* grew efficiently in *S. cerevisiae* strains W303 (*Sc*W303) and NB40 (*Sc*NB40), while strain BY (*Sc*BY) supported only a weak infection (Figure 1a). After 14 days the percentage of infected cells counted by FISH using probes against the *Wolbachia* 16S rDNA was 71.8% ± 8.7% for *Sc*W303 and 52.3% ± 14.3% for *Sc*NB40, while in *Sc*BY less than 20% cells were positive for FISH (Figure 1b). Strain *Sc*W303 was chosen for further studies. *Sc*W303 maintains high rate of oxidative phosphorylation regardless of the carbon source, it is highly resistant to oxidative stress (Ocampo, Liu, Schroeder, Shadel, & Barrientos, 2012) and it has a weak cell wall (Avrahami‐Moyal et al., 2012). Strains used in this study are detailed in Table S1.

### 3.2 Proliferation of *Wolbachia* in *Sc*W303 was further confirmed using different independent methods as follows (Figure 2)

**Figure 2:**
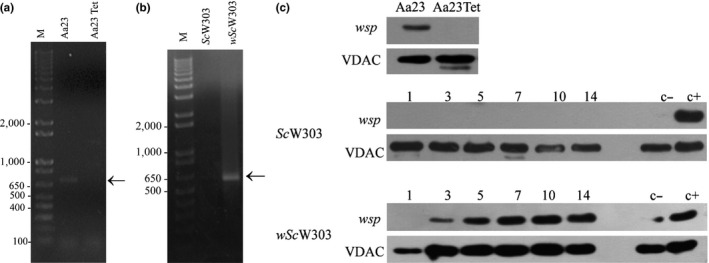
Detection of *Wolbachia* in *Saccharomyces cerevisiae* W303. (a) Agarose gel electrophoresis of the *wsp* PCR products predicted as a ~600 bp band in the Aa23 cell line. Lanes: M, Invitrogen 1 kb plus DNA ladder; Aa23, infected cell line; Aa23 Tet, noninfected cell line. (b) Agarose gel electrophoresis of the *wsp* PCR products for *S. cerevisiae*. Lanes: M, Invitrogen 1 kb plus DNA ladder; *Sc*W303, uninfected original yeast; *wSc*W303, infected yeast. (c) Western blot against the wsp and VDAC proteins. First row, first lane Aa23 infected cell line and second lane Aa23∆w tetracycline‐cured cell line. Yeast samples were taken at 1, 3, 5, 7, 10, and 14 days postinfection. A positive control was taken from a 20 day YPD‐2% agar culture infected‐*wSc*W303. Negative control was a noninfected sample *Sc*W303

#### 3.2.1 PCR of the *Wolbachia* outer surface protein gene (*wsp*)

Both the Aa23 cell line (Figure 2a) and infected *S. cerevisiae* (*wSc*W303) (Figure 2b) amplified 650 bp fragments exhibiting sequences 100% identical to the surface protein of the *Wolbachia* endosymbiont of *Aedes albopictus* (NCBI database: KC242223.1) (Table S2). PCR amplification bands were not observed in the tetracycline‐treated Aa23 cell line (Figure 2a, Aa23 Tet) and in the noninfected yeast (Figure 2b, *Sc*W303). Tetracycline used continuously in cell cultures is reported to kill *Wolbachia* (Dobson et al., 2002).

#### 3.2.2 Western Blot analysis detected *Wolbachia* wsp in *S. cerevisiae*

In the Aa23 cell line, a ~37 kDa protein corresponding to the *Wolbachia* Surface protein (wsp) was revealed with anti wsp antibodies (Bei resources, NIH, MD) (Figure 2c, Aa23). This band disappeared after growth in the presence of tetracycline (Aa23 Tet). VDAC (Voltage dependent anionic channel) protein was used as a loading control. In non–infected yeast wsp was not detected, (Figure 2c, *Sc*W303), while in infected yeast the wsp western blot signal was first detected at day 3 and increased gradually up to day 10, remaining stable until day 14 (Figure 2c, *wSc*W303). (For images of original Western Blots, see Figure S1a). When tetracycline was added to the medium, the wsp signal decreased, disappearing by day 10 (Figure S1b, *wSc*W303Tet).

#### 3.2.3 Normal growth and early death were observed in infected *S. cerevisiae*

**Figure 3:**
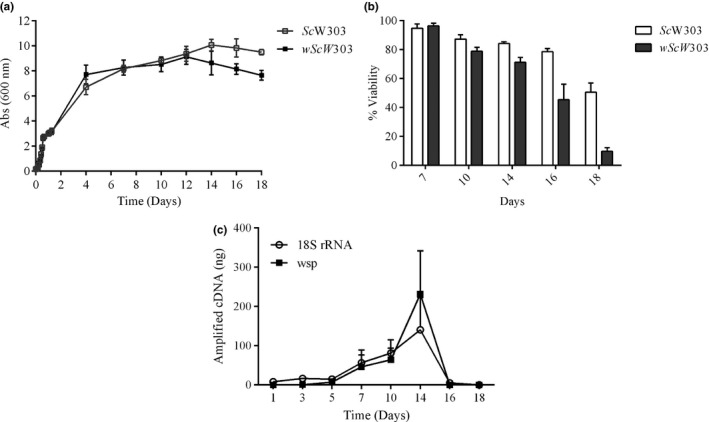
*S. cerevisiae* growth, viability, and transcriptional activity in the absence and presence of *Wolbachia*. (a) Growth curves of *Sc*W303 and *wSc*W303 grown in YPDS at 30°C, 130 rpm for 18 days. (b) Yeast cell viability in different days of culture quantified by microscopy with the BacLight viability kit. (c) Amplification of the *wsp* gene of *Wolbachia* and the 18S rRNA gene of *S. cerevisiae* of samples taken at different days of culture

During the first 12 days of culture, growth curves of infected *wSc*W303 were similar to the controls (Figure 3a). Then, beginning at day 14, *wSc*W303 absorbance decreased. Cell wall degradation (Figure S2) and viability staining (Figure 3b) confirmed that *wSc*W303 viability was rapidly lost during the late stages of the stationary phase, from 14 to 18 days of culture.

In addition, during growth the transcriptional activity of both *S. cerevisiae* 18S rRNA and the *Wolbachia wsp* were tested. Transcription was high in *S. cerevisiae* from the first day, decreased at day fourteen and became negligible at days 16 and 18 (Figure 3c). In contrast, transcription of the *wsp* from *Wolbachia* became detectable only after 3 days, increased exponentially until day 10 and remained constant until day 14. Then, at days 16 and 18, transcription decreased abruptly (Figure 3c). Transcription data in the *Wolbachia/S. cerevisiae* system indicated that *Wolbachia* activity grew later than *S. cerevisiae*, reaching a maximum at 10 days. Later, beginning at 14 days both transcription activities decreased abruptly in parallel with the death of the host.

#### 3.2.4 Yeast cell/endosymbiont images were observed by staining *S. cerevisiae* with Calcofluor white and *w*AlbB with Quasar 670

**Figure 4:**
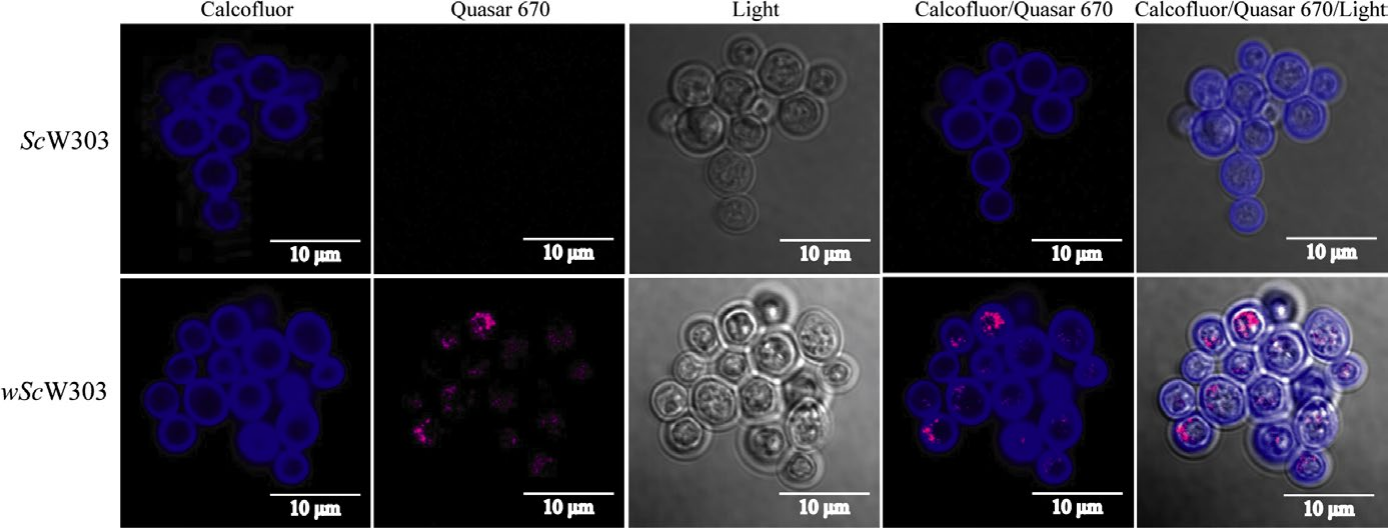
*Wolbachia* in calcofluor‐labeled *S. cerevisiae*. Infected (*wSc*W303) and noninfected (*Sc*W303) *S. cerevisiae* cells were hybridized with the 16S rDNA *Wolbachia* probe (Quasar 670, pink). Then, the yeast cell wall was stained with calcofluor‐white (Calcofluor, blue) to confirm the endosymbiosis. Merge images are shown to evaluate the presence of *Wolbachia* inside yeast

Both the western blot and the RT‐PCR experiments suggested that *Wolbachia* grew in the presence of *S. cerevisiae* becoming abundant at days 10–14. In order to determine whether *Wolbachia* was inside yeast, samples from infected and noninfected yeast cells from 14‐day old cultures were hybridized using a *Wolbachia* specific 16S rDNA probe labeled with Quasar‐670 (FISH). Then, the yeast cell wall was stained with Calcofluor white (Figure 4, Movie S1 ,MovieS2), Staining of the *S. cerevisiae* cell wall allowed observation of labeled bacteria inside yeast. Figure 4 shows that the Quasar‐670 label was detectable only in *wSc*W303 and not in *Sc*W303. Merge of the Calcofluor, Quasar‐670 and Clear field (Light) images show bacteria are inside the cell (Figure 4). Tridimensional reconstructions of z‐cuts performed in a *wSc*W303 sample show the intracellular location of different bacteria (movies S1 and S2). In the periphery of movies S1 and S2, few independent bacterial labels were detected, which we speculate, may come from bacteria inside heavily deteriorated host cells whose cell wall was not stained by Calcofluor (movies S1 and S2).

#### 3.2.5 TEM images detected *Wolbachia* inside *S. cerevisiae*

**Figure 5:**
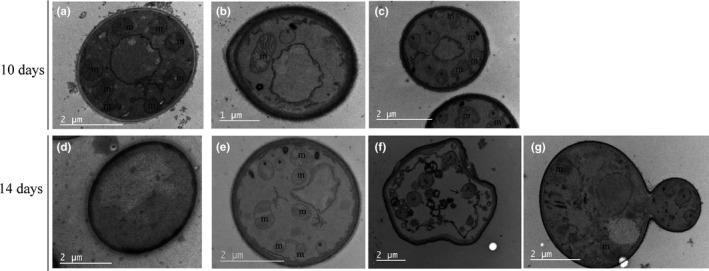
Electron microscopy images of infected and noninfected *Saccharomyces cerevisiae* at different times of incubation. Transmission electron microscopy images confirm the intracellular location of *Wolbachia*. 10‐day images were taken with uninfected (a) *Sc* and infected (b–c) *wSc*; 14 day‐old images were taken with (d) *Sc*W303 and (e–g) *wSc*W303. Images show the presence of bacteria‐like bodies (*) that are not present in uninfected yeast and mitochondria (m) whose cristae can be easily identified

Transmission electron microscopy images further suggested the intracellular location of *Wolbachia*. Cultures of 10 and 14 days of control and infected *S. cerevisiae* were analyzed. In infected yeast cells (Figure 5b–f, g), bacteria‐like bodies (Figure 5, labeled *) that are not present in the uninfected yeast (Figure 5a and d) can be observed. At 10 days both infected and noninfected yeast present mitochondria, which can be identified by the presence of inner membrane cristae (Figure 5, labeled m). In contrast 14 day‐old cultures of *Sc*W303 lost most mitochondrial structures, which suggest that these organelles are dysfunctional probably because cells are in late stationary phase. In contrast, 14 days‐old infected *wSc*W303 show *Wolbachia* plus mitochondria where the typical cristae pattern may be observed, suggesting abnormal preservation of mitochondria in infected yeast (Figure 5e–g). In *wSc*W303 cultures, we can observe different cell images: most cells had an intact plasma membrane and contained mitochondria and bacteria‐like bodies inside (Figure 5e). Other cells exhibited damaged membranes but the bacteria like structures were still present (Figure 5f). Among the whole population, we found some budding yeast, where bacteria‐like bodies can be seem concentrated in the bud (Figure 5g). None of the latter populations was found in control *Sc*W303 cultures.

#### 3.2.6 *Wolbachia‐*infected yeast retained high mitochondrial oxidative phosphorylation activity for abnormally long periods

A possible mechanism for the early death of infected yeast was explored in our infected *Sc*W303/*w*AlbB system. This system exhibited an abnormal preservation of mitochondria (Figure 5), so it was logical to explore aerobic metabolic activity. The relationship between *Wolbachia* and aerobic metabolism in the host is a matter of controversy. Some authors have proposed that these endo‐cellular organisms possess an aerobic metabolism that contributes to overall activity (Strübing et al., 2010) while others suggest that *Wolbachia* optimizes aerobic metabolism by supplying heme groups for respiratory complexes (Darby et al., 2012; Fallon, Baldridge, Carroll, & Kurtz, 2014; Foster et al., 2005; Heddi et al., 1999; Strübing et al., 2010). Thus, we decided to evaluate oxidative phosphorylation activities in our system, which preserved mitochondrial structure beyond the stationary phase (Figure 5).

When isolation of *Wolbachia* was attempted, it was found that the bacterium and mitochondria migrated together (Baldridge et al., 2014; Uribe et al., 1985). Thus, it was decided to characterize oxidative phosphorylation activity in the mitochondria/*Wolbachia* mixture and then determine the contribution of each entity using different bioenergetics techniques. The rate of oxygen consumption was measured using ethanol as a substrate (Table 1). We isolated the mitochondria/*Wolbachia* fraction from either one‐day cultures where there are very few *Wolbachia* cells or from 14‐day cultures, where *Wolbachia* numbers were high (Table 1). In one‐day cultures from *Sc*W303 and *wSc*W303 respiratory activities were very similar. However, at 14 days the rates of oxygen consumption and respiratory controls (RC) were widely different as follows: In noninfected yeast, both the rate of oxygen consumption and respiratory control decreased at the expense of state 3 inhibition, while in contrast, *wSc*W303 retained high rates of oxygen consumption plus high respiratory controls, i.e. in 14‐day old *Wolbachia*‐infected yeast exhibited high oxidative phosphorylation activity, consistent with the presence of mitochondria observed by TEM in the infected cells (Table 1).

**Table 1:** Oxygen consumption rates of mitochondrial fractions from 1 and 14 day‐old cultures of *Wolbachia*‐infected (*wSc*W303) and noninfected (*Sc*W303) *Saccharomyces cerevisiae* cells

	State IV (natgO/min*mg prot)	State III (natgO/min*mg prot)	RCIII/IV
1 Day *Sc*W303	25.2 ± 3.1	52.5 ± 6.8	2.1 ± 0.15
1 Day *wSc*W303	27.4 ± 4.6	65.4 ± 9.0	2.4 ± 0.2
14 Days *Sc*W303	22.6 ± 5.1	29.0 ± 6.3	1.3 ± 0.2
14 Days *wSc*W303	34.2 ± 5.1	73.3 ± 11.1	2.1 ± 0.1

Reaction mixture: 0.6 mol L^−1^ mannitol, 5 mmol L^−1^ MES, pH 6.8, 4 mmol L^−1^ Pi, 10 mmol L^−1^ KCl. As substrate, 5 mmol L^−1^ ethanol. For state III, 1 mmol L^−1^ ADP.

#### 3.2.7 In the presence of *Wolbachia* the activity of different mitochondrial respiratory complexes was preserved

**Figure 6:**
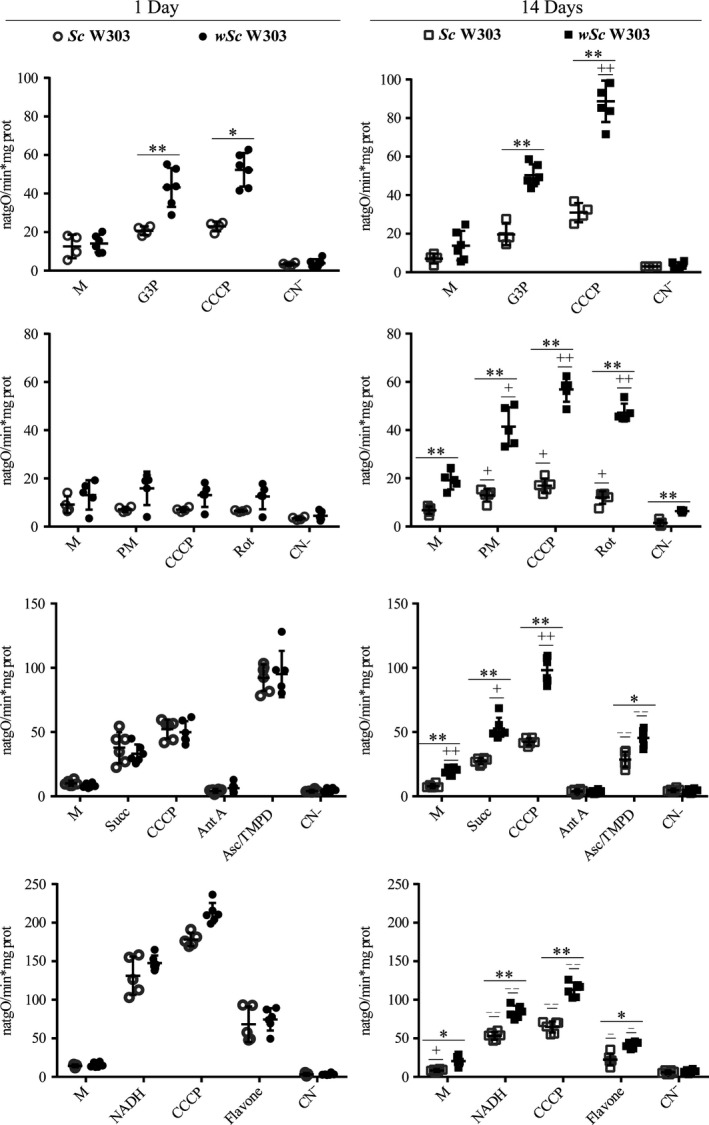
*Wolbachia*–mediated effects on the oxygen consumption activity of isolated yeast mitochondria. High‐resolution respirometry. 1 day‐old cultures of noninfected (1 Day *Sc*W303) and infected (1 Day *wSc*W303) yeast and 14 day‐old cultures of noninfected (14 Days *Sc*W303) and infected (14 Days *wSc*W303) yeast. 5 mmol L^−1^ from each substrate was added as indicated: glycerol‐3‐phosphate (G3P), NADH, pyruvate‐malate (Pyr‐Mal), succinate (Succ), and ascorbate‐TMPD (Asc/TMPD). Where indicated, 0.5 μmol L^−1^ CCCP, 0.1 μmol L^−1^ rotenone (Rot), 0.1 μmol L^−1^ antimycin A (Ant A), 1 mmol L^−1^ cyanide (CN‐), and 0.15 mmol L^−1^ flavone. 0.5 mg prot/ml of mitochondria (M) were added. Data represent mean ± SEM. T test *p < .005, *^*^ *p* < .001 for *Sc*W303 versus *wSc*W303 yeast on the same day. *T* test ^**−/+**^ *p* < .05 ^**− −**^^/^^**++**^ *p* < .001 (^**−**^, decrease; ^**+**^, increase) for *Sc*W303 in day one versus day 14 cultures or *wSc*W303 in day one versus day 14 cultures

In the isolated mitochondria/*Wolbachia* mixture, we tested specific substrates for each respiratory chain complex/enzyme (Table S3). In one‐day cultures the rates of oxygen consumption were similar in infected and noninfected *S. cerevisiae* (Figure 6). In aged mitochondria from noninfected yeast, external NADH dehydrogenase (NDH2e, Pyruvate‐Malate), succinate dehydrogenase (Succinate) and Complex IV (Ascorbate‐TMPD) activities were strongly diminished. In contrast, in the 14 day‐old mitochondrial fractions from *Wolbachia*‐infected cells, respiratory activities in the presence of glycerol‐3‐phosphate, pyruvate‐malate, and succinate were increased in comparison to 1‐day cultures. Since *S. cerevisiae* does not have complex I and pyruvate‐malate dependent respiration was insensitive to rotenone, redox activity was most likely from the mitochondrial NDH2 and not a bacterial complex I. Succinate oxidation was completely inhibited by antimycin A, indicating the absence of an alternative oxidase. Complex IV and NADH‐dependent oxygen consumption rates were still decreased as compared to mitochondria from one‐day cultures (Figure 6). Other respiratory substrates, namely glutamate and glutamine, which are used by Rickettsia (Winkler & Turco, 1988) where assayed and they did not support oxygen consumption. The respiratory activities measured indicate that the mitochondria/*Wolbachia* fractions from the infected and noninfected yeast consume the same substrates and are inhibited by the same respiratory chain inhibitors.

#### 3.2.8 Under the experimental conditions tested, infected *wSc*W303 oxygen consumption activity was mitochondrial

The experiments above suggested that either *Wolbachia* has the exact same electron transport chain as mitochondria or *Wolbachia* respiratory proteins may be damaged when the mitochondria/*Wolbachia* fraction is isolated and exposed to oxygen.

**Figure 7:**
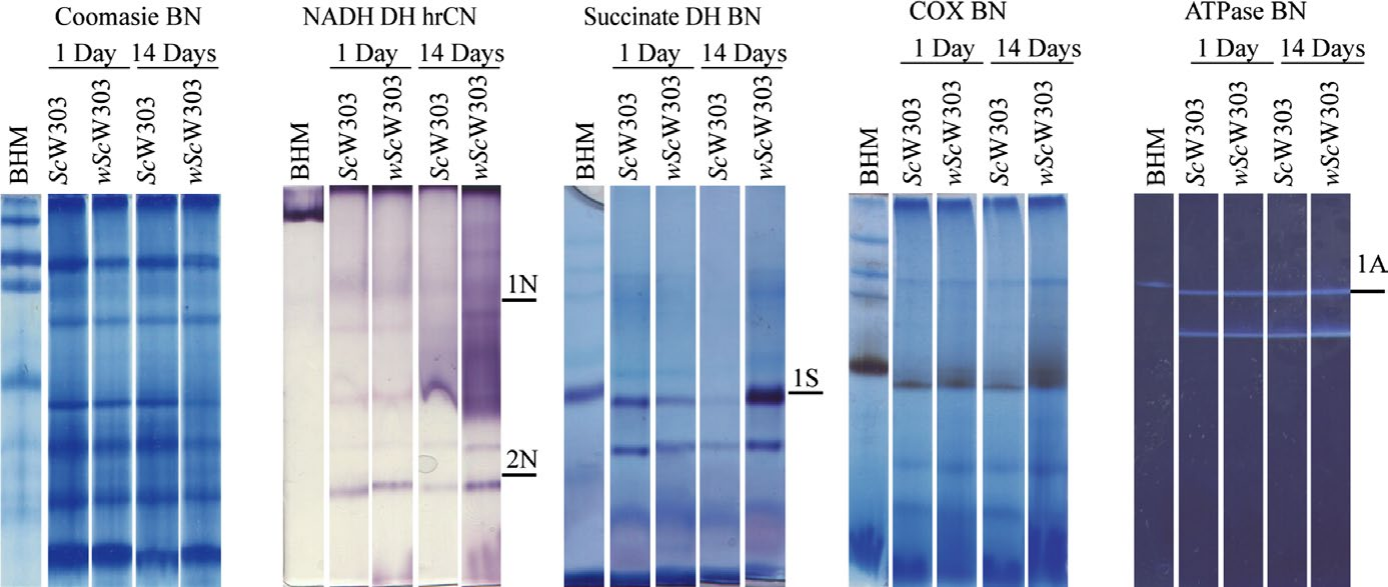
*Wolbachia*–mediated effects on the activity of each mitochondrial respiratory complex. BN‐PAGE Coomassie‐stained gel of *Sc*W303 and *wSc*W303 cells harvested at one and 14 days. In gel NADH‐NBT oxido‐reductase, succinate dehydrogenase, cytochrome *c* oxidase and ATPase enzyme activities in hrCN‐PAGE or BN‐PAGE, from 14 day old culture of *wSc*W303. Bands 1N, 2N, 1S, and 1A were sequenced (Table S4). Bovine heart mitochondria (BHM) were used as a control

To explore this possibility further, we measured in‐gel activities in the mitochondria/bacterium fraction. As eukaryote and prokaryote respiratory complexes I, II, III, and IV have different molecular masses the contribution from each organism to a given activity would be easily detected by native gel electrophoresis. The in‐gel activities for each complex from infected and noninfected yeast from 1 and 14 day‐old cultures were analyzed and, in all cases, activities were detected only at MWs corresponding to the mitochondrial enzymes (Table S3, Figure 7) suggesting that in the artificial *Sc*W303/*w*AlbB system and under the specific conditions of growth reported here, *Wolbachia* did not express any functional respiratory chain proteins. The above results suggest that mitochondria were responsible for all the observed oxygen consumption activity. Still, one NADH dehydrogenase (Table S4) was weakly expressed making it impossible to conclude on whether different *Wolbachia* strains may be aerobic or not.

#### 3.2.9 F_1_F_0_‐ATPase subunits from *Wolbachia* were detected in *wSc*W303

In the in gel ATPase activity from the mitochondria/*Wolbachia* isolate no differential bands were observed. This was expected as the proposed MWs are similar for of both ATPases: 543 kDa for the eukaryote *S. cerevisiae* and 530 kDa for prokaryotes *Escherichia coli* and *Paracoccus denitrificans* (Bakhtiari, Lai‐Zhang, Yao, & Mueller, 1999; Jonckheere, Smeitink, & Rodenburg, 2012; Morales‐Rios, Montgomery, Leslie, & Walker, 2015; Robinson et al., 2013; Schagger, 2002). However, the ATPase activity band (Figure 7A1,Table S4) sequence exhibited a mixture of yeast and *Wolbachia* ATPase proteins. BN and hrCN‐PAGE results indicate that if *Wolbachia* expresses any electron transport chain proteins (still a possibility), under our experimental conditions their concentration was negligible when compared to the mitochondrial proteins and to its own F_1_F_0_‐ATPase.

#### 3.2.10 *Wolbachia* remains infective against insect cell lines

**Figure 8:**
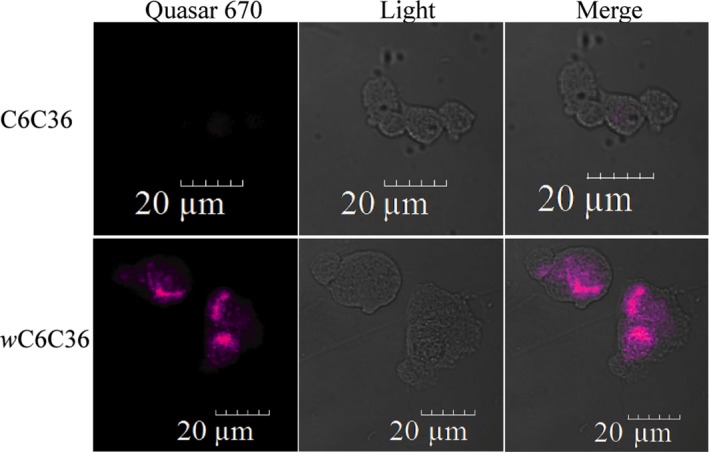
*Wolbachia* remains infective after being cultured in *S. cerevisiae*. FISH (Quasar 670‐pink) of the *w*C6C36 cell line. Light/Quasar images show hybridization only inside the infected cell line. The noninfected cell line C6C36 does not have any of the pink hybridization mark identified as *Wolbachia*

After being cultured in a yeast cell, the question arose on whether *Wolbachia* remained viable and infective when isolated. To test this, we extracted *Wolbachia* from *wSc*W303, incubated it in isolation for 5 days and then infected a C6C36 insect cell line which was previously reported to support bacterial infection (Baldridge et al., 2014). Aged *Wolbachia* infection was successful as assessed by specific staining using FISH (Figure 8Movie S3).

## 4 DISCUSSION

Growing obligate endosymbionts in cell lines yields low biomass at high costs (Baldridge et al., 2014; Khoo et al., 2013). To circumvent this problem, we built a synthetic host–endosymbiont system by artificially infecting the commonly used yeast *Saccharomyces cerevisiae* strain W303 with *Wolbachia w*AlbB from *A. albopictus* (Figures 1, 2, 3, 4, 5, Movies S1–S2). Culturing *Wolbachia* in yeast allowed us to study the complex relationship between the host and the bacterium. Using *S. cerevisiae* as an artificial host confers benefits such as a high resistance to changing environments (Gasch, 2002; Gasch & Werner‐Washburne, 2002), use of inexpensive liquid cultures and most importantly, the ease of manipulating and genetically engineering the host cell. Following our approach, it may be possible to construct other synthetic parasite‐mutualistic systems for obligate endosymbionts. Our system requirements for a successful infection were: supplementing YPD with iron and bovine fetal serum, plus low speed agitation of the nonbaffled Erlenmeyer flask and keeping the temperature between 28 and 30°C. These adjustments resulted in successful yeast infection and considerable *Wolbachia* yields in 14 days as compared with available methods that need up to 100 days. Even if the *Saccharomyces*/*Wolbachia* system is only a model of the interactions that occur in a naturally infected eukaryote cell, its manageability is outstanding and it may yield results that are not possible in cell lines.

Since *Wolbachia* is an alpha‐proteobacterium closely related to mitochondria, it seemed likely that the aerobic metabolic machinery of *Wolbachia* might mimic, enhance, or supplement the respiratory activity from the host. (Strübing et al., 2010). However, under our conditions, *Wolbachia* respiratory chain proteins were not detectable, instead, we found an increase in host mitochondrial activity. Another obligate endosymbiont, the *Sytophilus oryzae* Principal Endosymbiont (SOPE), has also been reported to increase the mitochondrial activity in the host, probably by providing nutrients such as riboflavin (Heddi, Lefebvre, & Nardon, 1993; Heddi et al., 1999). Several authors suggest that *Wolbachia* provides riboflavin or heme groups to their arthropod and nematode hosts (Brownlie et al., 2009; Darby et al., 2012; Foster et al., 2005; Wu et al., 2009). This may vary with strains as *Wolbachia* from *Brugia malayi* (*wBm)* contains complete sets of riboflavin, heme and nucleotide biosynthesis genes the filarial host lacks (Darby et al., 2012; Foster et al., 2005; Klasson et al., 2008; Wu et al., 2004). In return, the host provides amino acid, proteins and a safe, stable environment (Brownlie et al., 2009; Darby et al., 2012; Foster et al., 2005; Wu et al., 2009). The possibility that *Wolbachia*, behaves as SOPE, donating riboflavin or heme groups to the host may be explored in auxotrophic yeast mutants. *S. cerevisiae* libraries have a mutant for almost every enzyme on the riboflavin and heme synthesis pathways e.g. *S. cerevisiae* genome database (https://www.yeastgenome.org/).

Under the conditions tested here, expression of *Wolbachia* electron transport proteins was not detected. The reported *Wolbachia pipientis w*AlbB genome (Mavingui et al., 2012) shows that some respiratory complex subunits are missing, e.g. *nuo*C and *nuo*D for a functional complex I (Sazanov, 2015); yet other *Wolbachia* sequenced genomes contain all the genes necessary for a functional electron transport chain (Klasson et al., 2008), so maybe under different growth conditions, hosts and *Wolbachia* strains, bacterial respiratory proteins may be detected. It is suggested that other *Wolbachia* strains should be tested in order to determine whether some consume oxygen.

In our hands, *Wolbachia* infection resulted in activation of mitochondria beyond the stationary growth phase. It may be speculated that such activation constitutes an advantage for *Wolbachia* either due to quenching of oxygen in the cytoplasm (Rosas‐Lemus et al., 2016) or because *Wolbachia* needs high ATP that an active mitochondria provides (Potter, Badder, Hoade, Johnston, & Morten, 2016). It has already been suggested by experiments using paraquat that *Wolbachia* sensitivity to free radicals is higher than that of the host (Fallon et al., 2013) and it cannot survive outside a host cell unless it is kept in a 5% CO_2_ atmosphere (Rasgon et al., 2006). Also, high agitation speeds, which would increase oxygen concentrations, lead to loss of the *Wolbachia* infection (Result not shown). Thus, it is possible that *Wolbachia* enters the cytoplasm to hide from high atmospheric oxygen and then it optimizes cell metabolism to both, use host metabolites and find low cytoplasmic oxygen concentrations. Avoidance, i.e. hiding from oxygen, is a common behavior in oxyconformers (Rosas‐Lemus et al., 2016). In air, oxygen saturation concentration is ~21% (200 μmol L^−1^) while intracellular oxygen concentration ranges between 13.2% and 14% (126–133 μmol L^−1^) for rhabdomyosarcoma (RD) cells (Potter et al., 2016) or HEK293T cells (Abcam, 2014). When cells are exposed to lower ambient oxygen, intracellular oxygen concentration is also decreased: HEK293T cells exposed to 6% oxygen (50 μmol L^−1^) have an intracellular oxygen concentration below 2% (19 μmol L^−1^) (Abcam, 2014); RD cells exposed to 10% or 5% ambient oxygen reduce their intracellular oxygen concentration to 5.4% and 2.1% respectively (Potter et al., 2016). In addition, there is an intracellular oxygen gradient in the area surrounding the mitochondria in rat heart and hepatocytes, where oxygen concentration ranges between 3 (Gnaiger, 2003) and 6 μmol L^−1^ (Jones & Kennedy, 1982; Tamura, Oshino, Chance, & Silver, 1978). Thus, a mitochondrion‐containing host such as cell lines and yeast would probably provide the endosymbiont with a microaerobic environment. The mechanism for the increase in host mitochondrial activity needs to be defined.

In conclusion, we describe the infection of *S. cerevisiae* strain W303 by *Wolbachia w*AlbB. Infection led to premature death of the host and to an abnormal pattern of oxygen consumption. Further experiments using other yeast and other *Wolbachia* strains are needed to further explore oxidative phosphorylation patterns in the host/endosymbiont relationship. This system holds a large potential for different evaluations of biochemical and genetical processes in *Wolbachia*. Large biofermentors may be used to yield large amounts of biomass as required for different genomics and proteomics studies.

## CONFLICT OF INTEREST

None declared.
