# Supplementary figures and images for: Wolbachia pipientis grows in Saccharomyces cerevisiae evoking early death of the host and deregulation of mitochondrial metabolism
Source: Microbiologyopen. 2018 Jun 13;8(4):e00675. doi: 10.1002/mbo3.675 (PMC6460262; doi:10.1002/mbo3.675)

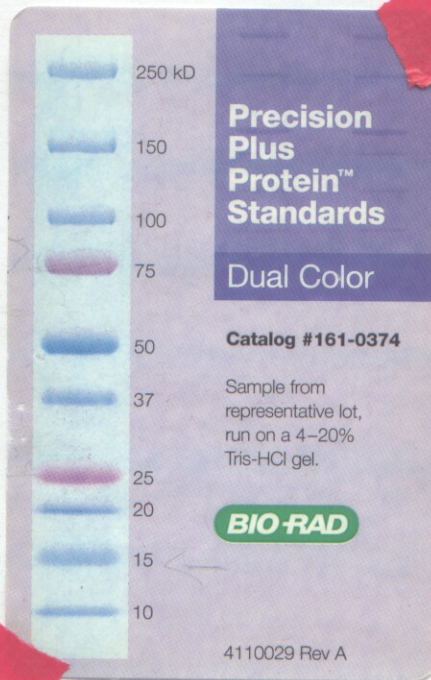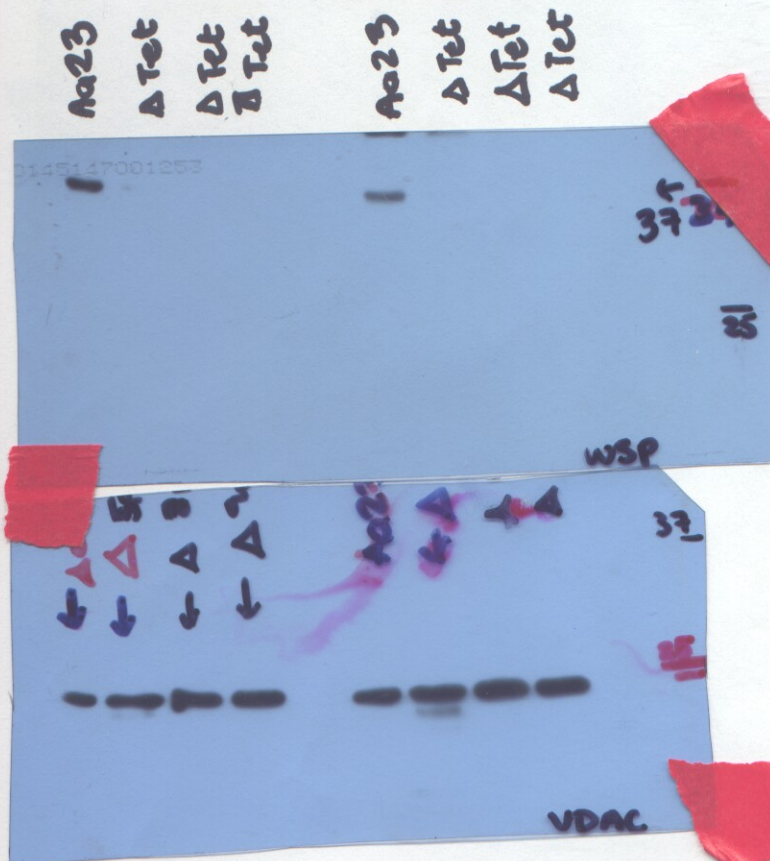

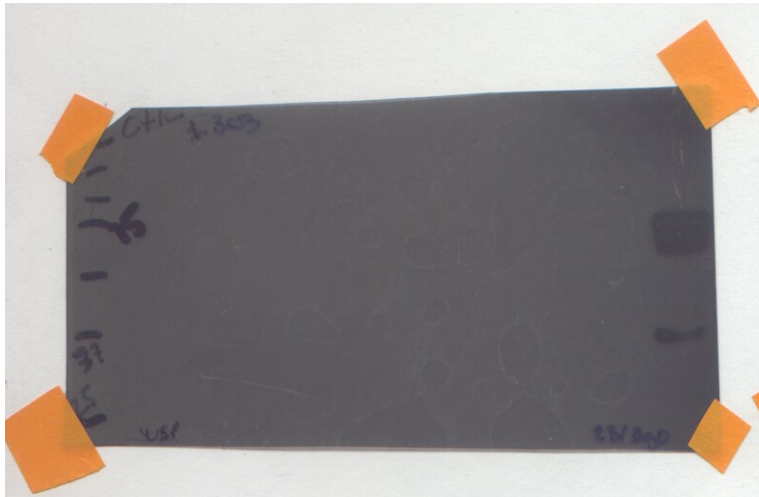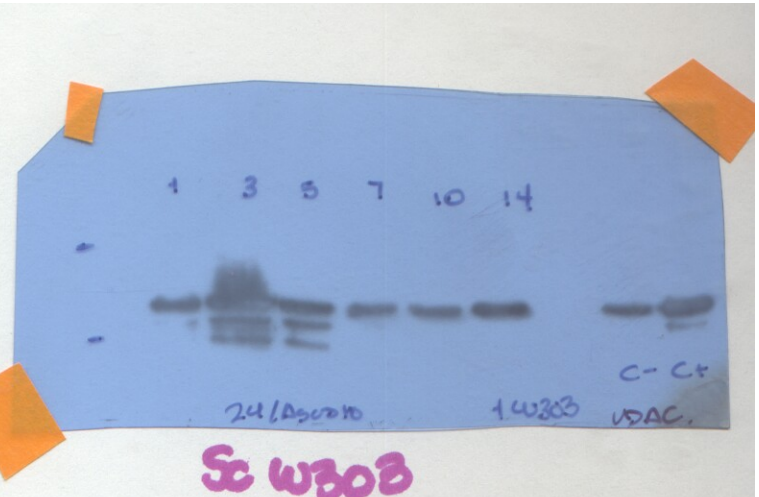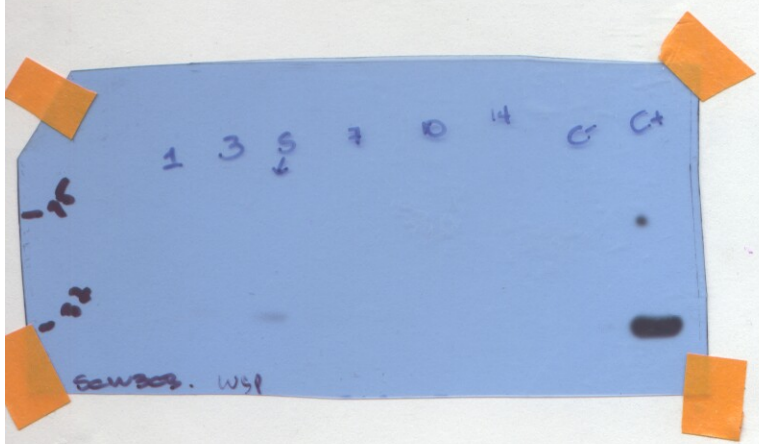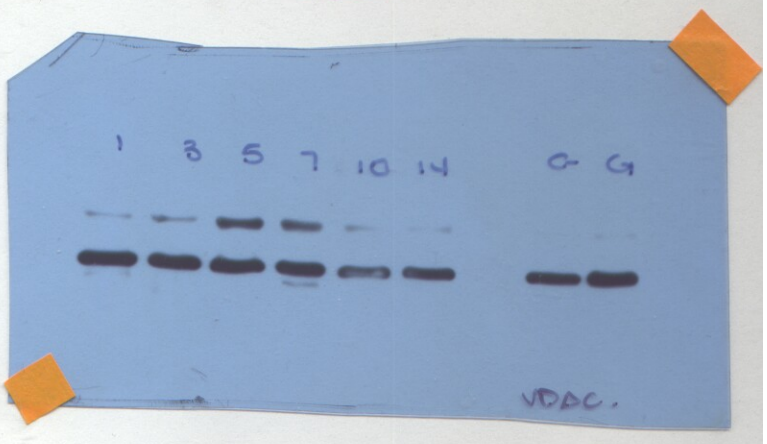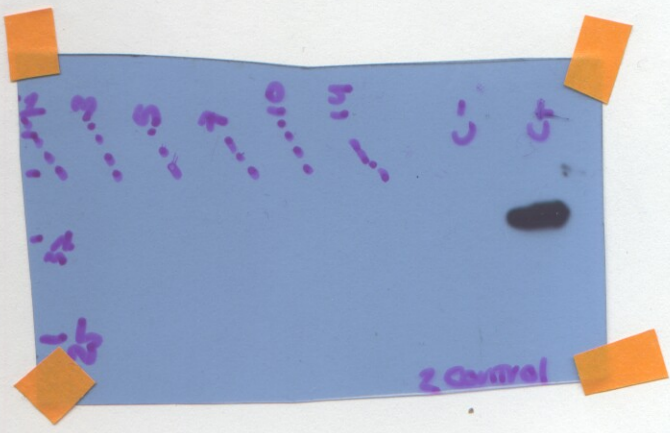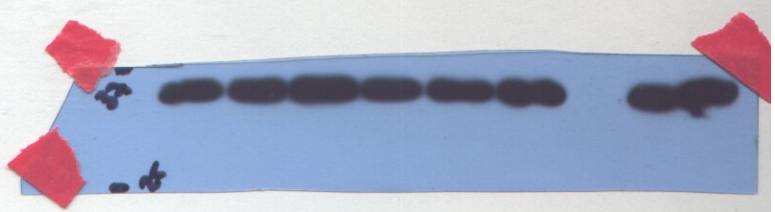

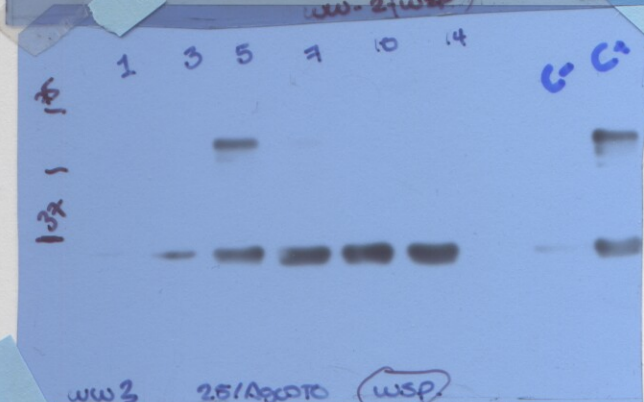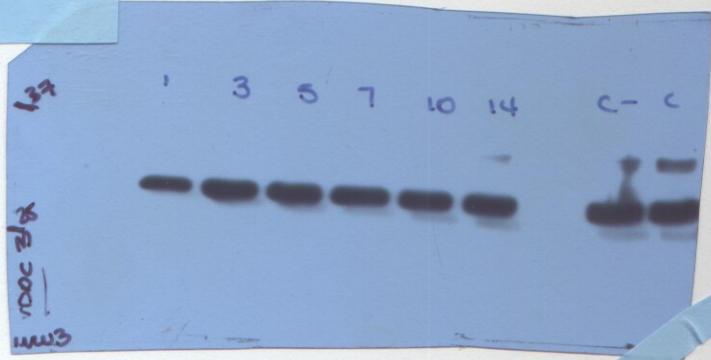

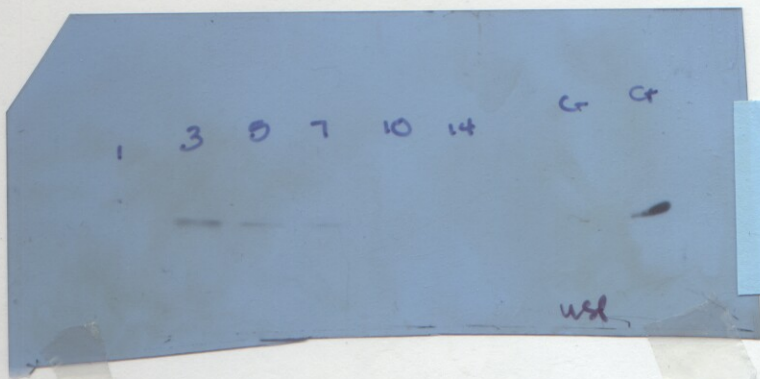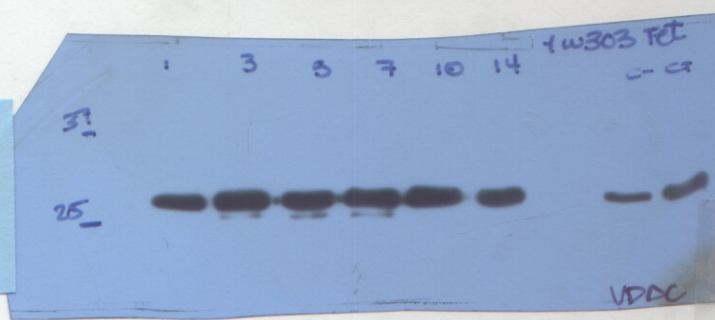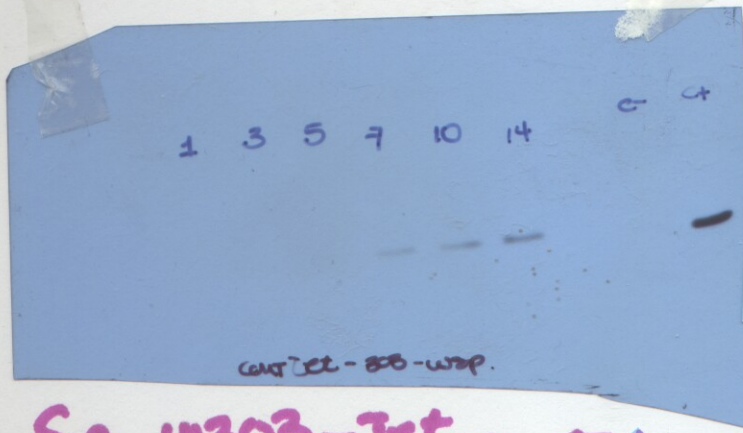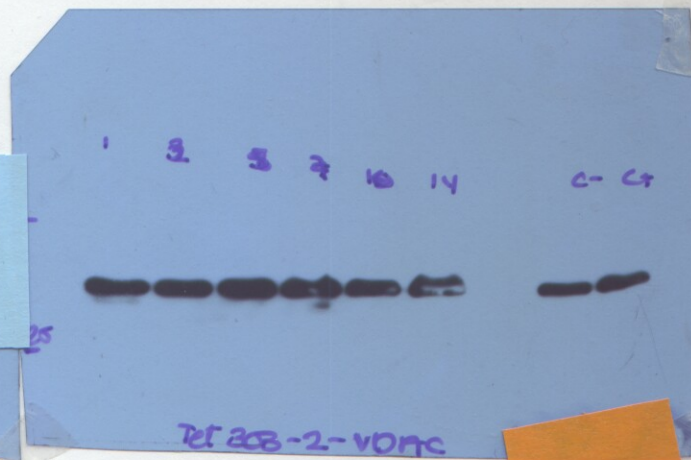

Sc w303-Tet (control)

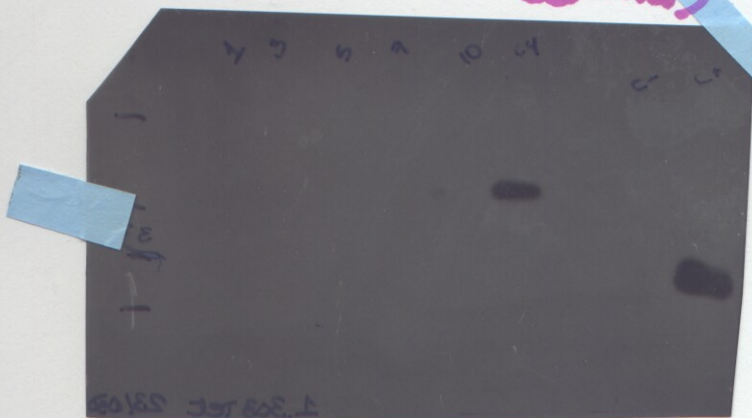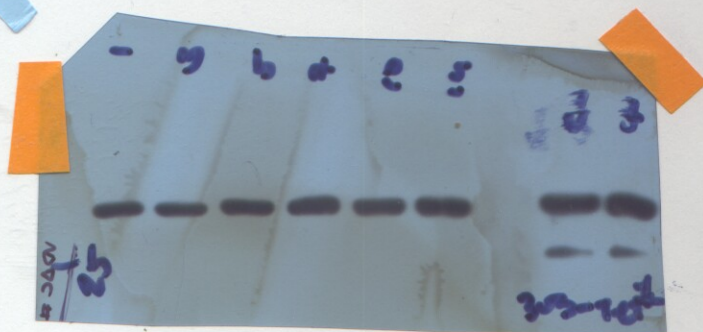

Infected + Tet.

WScW303 1eb.

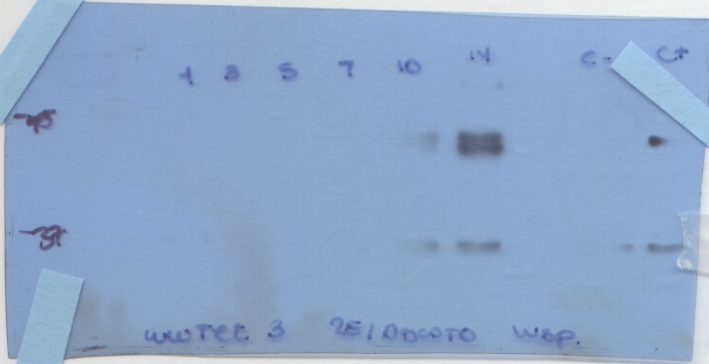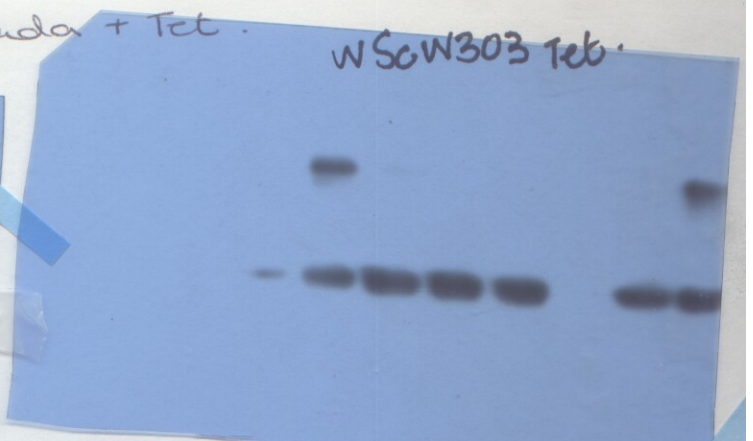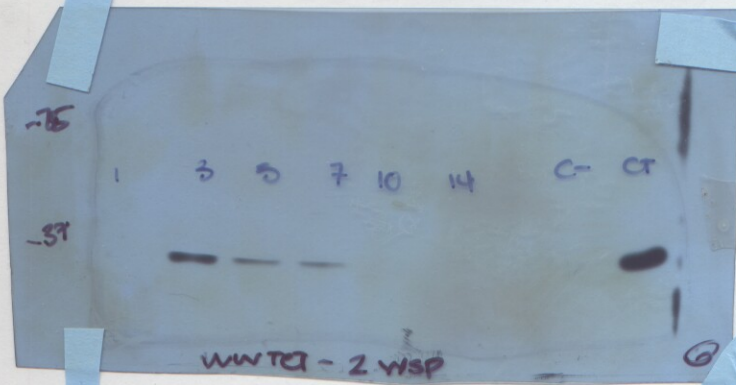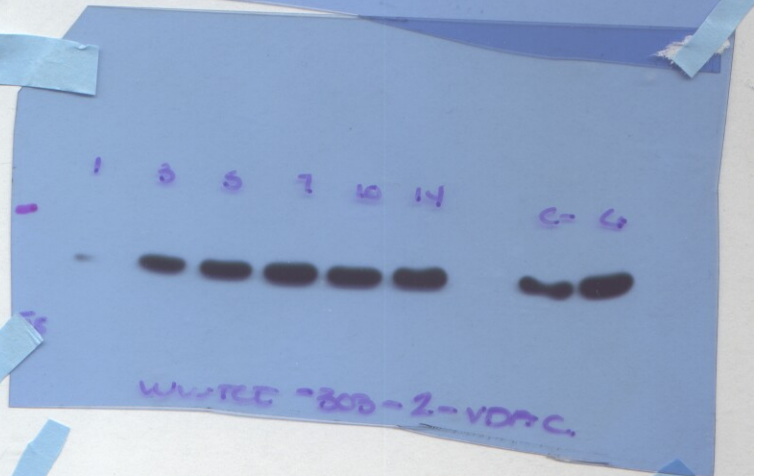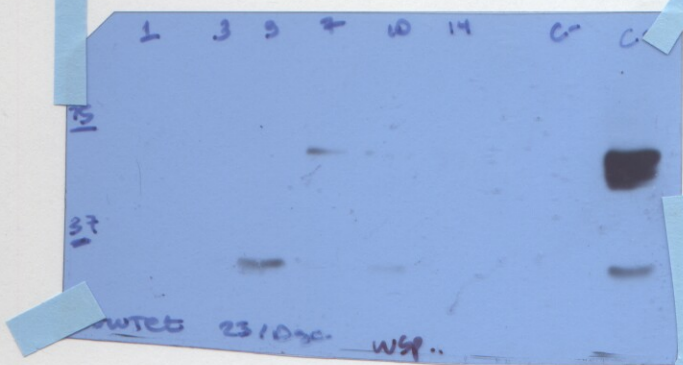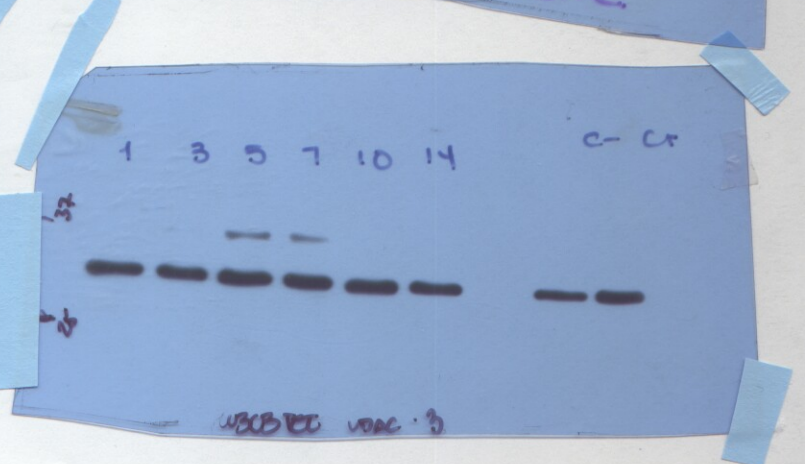

Supplement: Supplementary file 1 [file MBO3-8-e00675-s001.pdf]

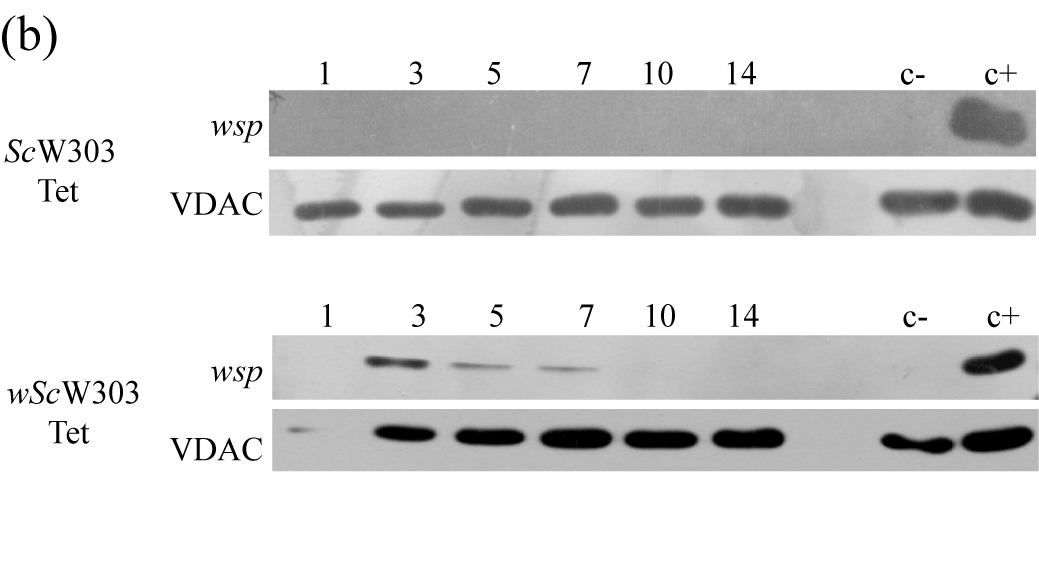

Supplement: Supplementary file 2 [file MBO3-8-e00675-s002.tif]
