# Supplementary material for: Wolbachia pipientis grows in Saccharomyces cerevisiae evoking early death of the host and deregulation of mitochondrial metabolism
Source: Microbiologyopen. 2018 Jun 13;8(4):e00675. doi: 10.1002/mbo3.675 (PMC6460262; doi:10.1002/mbo3.675)
Supplement: Supplementary file 4 [file MBO3-8-e00675-s004.docx]

Table S1. Yeast and *Wolbachia* strains used in this study.

| Strains | Reference |
| --- | --- |
| Aa23 cell line infected with *Wolbachia* *w*AlbB from Aedes albopictus. | (O´Neill, 1997) |
| *Saccharomyces cerevisiae* W303 (MATα; ura3-1; trp1Δ 2; leu2-3,112; his3-11,15; ade2-1; can1-100). | [(Gutierrez-Aguilar](#_ENREF_1) et al., 2014) |
| *Saccharomyces cerevisiae* BY4741 (MATa; his3 Δ1; leu2 Δ0; met15 Δ0; ura3 Δ). | [(Gutierrez-Aguilar](#_ENREF_1) et al., 2014) |
| *Saccharomyces cerevisiae* NB40-36A ((MATα; leu2, arg8::hisG, ura3-52; leu2-3,112). | (Pérez-Martínez et al., 2003) |
