# Supplementary material for: Wolbachia pipientis grows in Saccharomyces cerevisiae evoking early death of the host and deregulation of mitochondrial metabolism
Source: Microbiologyopen. 2018 Jun 13;8(4):e00675. doi: 10.1002/mbo3.675 (PMC6460262; doi:10.1002/mbo3.675)
Supplement: Supplementary file 5 [file MBO3-8-e00675-s005.docx]

**Supplementary table 1. Amplified PCR sequences**

| *Wolbachia* from Aa23 cell line *wsp* gene | Wsp691 | GTTGAtCTCTTTAGTAGCTGATACTGTTTCTTTATTAAAACTAGCACCATAAGAACCAAAATAACGAGCACCAGCATAAAGCTTGATTTCTGGGGTTACATCATAACTAACACCAGCTTTTGCTTGATAAGCAAAACCAAATCCTTTTTGATCTTTAACTGCACTAGCTTCTGAAGGATTGCTGATATATGCTGCACCAACACCAACACCAACGTATGGAGTGATAGGCATATCTTCAATCGCTATATCGTAATAAACGTTAACCAATCCTGAAAATACTGCCACACTGTTTGCAACAGTTGTTGGAGCAAATGTTGCACCACCAACGTCGTTTTTGTTTAGTTGTGAGTAAAGTCCCTCAACATCAACCCTGATATCGTCCATTTTATAACCAAATGCAGCACCACCAGCCATAAAAGATGCTTTTAAAGGATCATGAACTTCGGTTCCTTTTTTATATTCAATGCCGTCAATTCTTGTTTTAAAAGGTAAAACTTCACCATTATATTGCAAACGAACATAGTAGCTAGTTTCTTCATCACT |
| --- | --- | --- |
| *Wolbachia* from Aa23 cell line *wsp* gene | Wsp81 | NTNGCATANATGGTGAAGTTTTACCTTTTAAAACAAGAATTGACGGCATTGAATATAAAAAAGGAACCGAAGTTCATGATCCTTTAAAAGCATCTTTTATGGCTGGTGGTGCTGCATTTGGTTATAAAATGGACGATATCAGGGTTGATGTTGAGGGACTTTACTCACAACTAAACAAAAACGACGTTGGTGGTGCAACATTTGCTCCAACAACTGTTGCAAACAGTGTGGCAGTATTTTCAGGATTGGTTAACGTTTATTACGATATAGCGATTGAAGATATGCCTATCACTCCATACGTTGGTGTTGGTGTTGGTGCAGCATATATCAGCAATCCTTCAGAAGCTAGTGCAGTTAAAGATCAAAAAGGATTTGGTTTTGCTTATCAAGCAAAAGCTGGTGTTAGTTATGATGTAACCCCAGAAATCAAGCTTTATGCTGGTGCTCGTTATTTTGGTTCTTATGGTGCTAGTTTTAATAAAGAAACAGTATCAGCTACTAAAGAGATCAACGTTCTTTACAGCGCTGTTGGTGCAGAAGCTGGA |
| Infected *Saccharomyces cerevisiae* W303 *wsp* gene | Wsp691 | TTGANCTCTTTAGTAGCTGATACTGTTTCTTTATTAAAACTAGCACCATAAGAACCAAAATAACGAGCACCAGCATAAAGCTTGATTTCTGGGGTTACATCATAACTAACACCAGCTTTTGCTTGATAAGCAAAACCAAATCCTTTTTGATCTTTAACTGCACTAGCTTCTGAAGGATTGCTGATATATGCTGCACCAACACCAACACCAACGTATGGAGTGATAGGCATATCTTCAATCGCTATATCGTAATAAACGTTAACCAATCCTGAAAATACTGCCACACTGTTTGCAACAGTTGTTGGAGCAAATGTTGCACCACCAACGTCGTTTTTGTTTAGTTGTGAGTAAAGTCCCTCAACATCAACCCTGATATCGTCCATTTTATAACCAAATGCAGCACCACCAGCCATAAAAGATGCTTTTAAAGGATCATGAACTTCGGTTCCTTTTTTATATTCAATGCCGTCAATTCTTGTTTTAAAAGGTAAAACTTCACCATTATATTGCAAACGAACATAGTAGCTAGTTTCTTCATCACTAATTGACGTTGACTCTTTAGTAGCTGATACTGTTTCTTTATTAAAACTAGCACCATAAGAACCAAAATAACGAGCACCAGCATAAAGCTTGATTTCTGGGGTTACATCATAACTAACACCAGCTTTTGCTTGATAAGCAAAACCAAATCCTTTTTGATCTTTAACTGCACTAGCTTCTGAAGGATTGCTGATATATGCTGCACCAACACCAACACCAACGTATGGAGTGATAGGCATATCTTCAATCGCTATATCGTAATAAACGTTAACCAATCCTGAAAATACTGCCACACTGTTTGCAACAGTTGTTGGAGCAAATGTTGCACCACCAACGTCGTTTTTGTTTAGTTGTGAGTAAAGTCCCTCAACATCAACCCTGATATCGTCCATTTTATAACCAAATGCAGCACCACCAGCCATAAAAGATGCTTTTAAAGGATCATGAACTTCGGTTCCTTTTTTATATTCAATGCCGTCAATTCTTGTTTTAAAAGGTAAAACTTCACCATTATATTGCAAACGAACATAGTAGCTAGTTTCTTCATCACTNAA |
| Infected *Saccharomyces cerevisiae* W303 *wsp* gene | Wsp 81 | TANATGGTGAAGTTTTACCTTTTAAAACAAGAATTGACGGCATTGAATATAAAAAAGGAACCGAAGTTCATGATCCTTTAAAAGCATCTTTTATGGCTGGTGGTGCTGCATTTGGTTATAAAATGGACGATATCAGGGTTGATGTTGAGGGACTTTACTCACAACTAAACAAAAACGACGTTGGTGGTGCAACATTTGCTCCAACAACTGTTGCAAACAGTGTGGCAGTATTTTCAGGATTGGTTAACGTTTATTACGATATAGCGATTGAAGATATGCCTATCACTCCATACGTTGGTGTTGGTGTTGGTGCAGCATATATCAGCAATCCTTCAGAAGCTAGTGCAGTTAAAGATCAAAAAGGATTTGGTTTTGCTTATCAAGCAAAAGCTGGTGTTAGTTATGATGTAACCCCAGAAATCAAGCTTTATGCTGGTGCTCGTTATTTTGGTTCTTATGGTGCTAGTTTTAATAAAGAAACAGTATCAGCTACTAAAGAGATCAACGTTCTTTACAGCGCTGTTGGTGCAGAAGCTGGATGCATANATGGTGAAGTTTTACCTTTTAAAACAAGAATTGACGGCATTGAATATAAAAAAGGAACCGAAGTTCATGATCCTTTAAAAGCATCTTTTATGGCTGGTGGTGCTGCATTTGGTTATAAAATGGACGATATCAGGGTTGATGTTGAGGGACTTTACTCACAACTAAACAAAAACGACGTTGGTGGTGCAACATTTGCTCCAACAACTGTTGCAAACAGTGTGGCAGTATTTTCAGGATTGGTTAACGTTTATTACGATATAGCGATTGAAGATATGCCTATCACTCCATACGTTGGTGTTGGTGTTGGTGCAGCATATATCAGCAATCCTTCAGAAGCTAGTGCAGTTAAAGATCAAAAAGGATTTGGTTTTGCTTATCAAGCAAAAGCTGGTGTTAGTTATGATGTAACCCCAGAAATCAAGCTTTATGCTGGTGCTCGTTATTTTGGTTCTTATGGTGCTAGTTTTAATAAAGAAACAGTATCAGCTACTAAAGAGATCAACGTTCTTTACAGCGCTGTTGGTGCAGAAGCTGGANN |
