# Supplementary material for: Wolbachia pipientis grows in Saccharomyces cerevisiae evoking early death of the host and deregulation of mitochondrial metabolism
Source: Microbiologyopen. 2018 Jun 13;8(4):e00675. doi: 10.1002/mbo3.675 (PMC6460262; doi:10.1002/mbo3.675)
Supplement: Supplementary file 6 [file MBO3-8-e00675-s006.docx]

**Table S3. Respiratory chain component from mammals, yeast and bacteria.**

|  | Specie | T/M | Molec. weight (kDa) | Prosthetic groups | Inhibitors | Ref. |
| --- | --- | --- | --- | --- | --- | --- |
| C I | Mammals (BHM) | 43/7 | 1000 | FMN/ 7(Fe-S) | Rotenone, amital, pierdicine A, acetogenins, etc. | ([Walker, 1992](#_ENREF_22); [Degli Esposti, 1998](#_ENREF_4); [Carroll et al., 2003](#_ENREF_2); [Zhu et al., 2016](#_ENREF_29)) |
|  | *Y. lipolytica* | 42/7 | 960 |  |  | ([Kerscher et al., 2001](#_ENREF_10); [Zickermann et al., 2015](#_ENREF_31)) |
|  | *E. coli*,  *T. thermophilus*,  *P. denitrificans* | 13-14 | 550 |  |  | ([Zickermann et al., 2000](#_ENREF_30); [Stolpe and Friedrich, 2004](#_ENREF_18); [Sazanov and Hinchliffe, 2006](#_ENREF_15); [Sazanov, 2015](#_ENREF_14)) |
| C II | Mammals (BHM) | 4 | 123 | FAD/ 3(Fe-S)/haem type *b* | Malonate, malate, oxaloacetate,  carboxine. | ([Hagerhall, 1997](#_ENREF_6); [Sun et al., 2005](#_ENREF_19); [Wittig et al., 2010](#_ENREF_24)) |
|  | *S. cerevisiae* | 4 | 120 |  |  | ([Hagerhall, 1997](#_ENREF_6)) |
|  | *E. coli* | 4 | 360 (T) |  |  | ([Cecchini, 2003](#_ENREF_3); [Yankovskaya et al., 2003](#_ENREF_27)) |
| C III | Mammals (BHM) | 11/1 | 482 (D) | 2 haem type *b* (*b_H_*-*b_L_*)/ 1 haem, *c1* y 1 Fe-S | Antimicine A, mixotiazol | ([Trumpower, 1990](#_ENREF_20); [Xia et al., 1997](#_ENREF_25); [Iwata et al., 1998](#_ENREF_8); [Zhang et al., 1998](#_ENREF_28); [Wittig et al., 2010](#_ENREF_24)) |
|  | *Y. lipolytica* | 9/1 | 458 (D) |  |  | ([Lange and Hunte, 2002](#_ENREF_11); [Solmaz and Hunte, 2008](#_ENREF_17)) |
|  | *P. denitrificans* | 3 | 122 (M) |  |  | ([Yang and Trumpower, 1986](#_ENREF_26)) |
| C IV | Mammals (BHM) | 13/3 | 205 (M) | 2 haem: *a* y *a3*/ 2 copper CuA y CuB | Cyanide,CO, NO, NO_2_. | ^(^[^Tsukihara et al., 1996^](#_ENREF_21)^)^ |
|  | *S. cerevisiae* | 11/3 | 189 (M) |  |  | ([Geier et al., 1995](#_ENREF_5)) |
|  | *P. denitrificans* | 14 | 130(M) |  |  | ([Iwata, 1998](#_ENREF_7); [Schagger, 2002](#_ENREF_16)) |
| C V | Mammals (BHM, human) | 16/2 | 597 (M) | - | Oligomicine | ([Watt et al., 2010](#_ENREF_23); [Jonckheere et al., 2012](#_ENREF_9)) |
|  | *S. cerevisiae* | 16/2 | 543 (M) |  |  | ([Bakhtiari et al., 1999](#_ENREF_1); [Jonckheere et al., 2012](#_ENREF_9); [Robinson et al., 2013](#_ENREF_13)) |
|  | *E. coli*  *P. denitrificans* | 8 | 530 (M) |  |  | ([Schagger, 2002](#_ENREF_16); [Jonckheere et al., 2012](#_ENREF_9); [Morales-Rios et al., 2015](#_ENREF_12)) |

Abbreviations: T/M,Total subunits/mitochondrial genome coded subunits; M, monomer; D, dimer; T, trimer; FMN, flavin mononucleotide; FAD, flavin adenin dinucleotide; CO, carbon monoxide; NO, nitogen monoxide; NO_2_, nitrogen dioxide. BHM (Bovine heart mitochondria), *Y. lipolytica* (*Yarrowia lipolytica*), *E. coli* (*Escherichia coli*), *T. thermophilus* (*Thermus thermophilus*), *P. denitrificans* (*Paracoccus denitrificans*), *S. cerevisiae* (*Saccharomyces cerevisiae*).

References.

Bakhtiari, N., et al. (1999). "Structure/function of the beta-barrel domain of F1-ATPase in the yeast Saccharomyces cerevisiae." J Biol Chem **274**(23): 16363-16369.

Carroll, J., et al. (2003). "Analysis of the subunit composition of complex I from bovine heart mitochondria." Mol Cell Proteomics **2**(2): 117-126.

Cecchini, G. (2003). "Function and structure of complex II of the respiratory chain." Annu Rev Biochem **72**: 77-109.

Degli Esposti, M. (1998). "Inhibitors of NADH-ubiquinone reductase: an overview." Biochim Biophys Acta **1364**(2): 222-235.

Geier, B. M., et al. (1995). "Kinetic properties and ligand binding of the eleven-subunit cytochrome-c oxidase from Saccharomyces cerevisiae isolated with a novel large-scale purification method." Eur J Biochem **227**(1-2): 296-302.

Hagerhall, C. (1997). "Succinate: quinone oxidoreductases. Variations on a conserved theme." Biochim Biophys Acta **1320**(2): 107-141.

Iwata, S. (1998). "Structure and function of bacterial cytochrome c oxidase." J Biochem **123**(3): 369-375.

Iwata, S., et al. (1998). "Complete structure of the 11-subunit bovine mitochondrial cytochrome bc1 complex." Science **281**(5373): 64-71.

Jonckheere, A. I., et al. (2012). "Mitochondrial ATP synthase: architecture, function and pathology." J Inherit Metab Dis **35**(2): 211-225.

Kerscher, S., et al. (2001). "Exploring the catalytic core of complex I by Yarrowia lipolytica yeast genetics." J Bioenerg Biomembr **33**(3): 187-196.

Lange, C. and C. Hunte (2002). "Crystal structure of the yeast cytochrome bc1 complex with its bound substrate cytochrome c." Proc Natl Acad Sci U S A **99**(5): 2800-2805.

Morales-Rios, E., et al. (2015). "Structure of ATP synthase from Paracoccus denitrificans determined by X-ray crystallography at 4.0 A resolution." Proc Natl Acad Sci U S A **112**(43): 13231-13236.

Robinson, G. C., et al. (2013). "The structure of F(1)-ATPase from Saccharomyces cerevisiae inhibited by its regulatory protein IF(1)." Open Biol **3**(2): 120164.

Sazanov, L. A. (2015). "A giant molecular proton pump: structure and mechanism of respiratory complex I." Nat Rev Mol Cell Biol **16**(6): 375-388.

Sazanov, L. A. and P. Hinchliffe (2006). "Structure of the hydrophilic domain of respiratory complex I from Thermus thermophilus." Science **311**(5766): 1430-1436.

Schagger, H. (2002). "Respiratory chain supercomplexes of mitochondria and bacteria." Biochim Biophys Acta **1555**(1-3): 154-159.

Solmaz, S. R. and C. Hunte (2008). "Structure of complex III with bound cytochrome c in reduced state and definition of a minimal core interface for electron transfer." J Biol Chem **283**(25): 17542-17549.

Stolpe, S. and T. Friedrich (2004). "The Escherichia coli NADH:ubiquinone oxidoreductase (complex I) is a primary proton pump but may be capable of secondary sodium antiport." J Biol Chem **279**(18): 18377-18383.

Sun, F., et al. (2005). "Crystal structure of mitochondrial respiratory membrane protein complex II." Cell **121**(7): 1043-1057.

Trumpower, B. L. (1990). "Cytochrome bc1 complexes of microorganisms." Microbiol Rev **54**(2): 101-129.

Tsukihara, T., et al. (1996). "The whole structure of the 13-subunit oxidized cytochrome c oxidase at 2.8 A." Science **272**(5265): 1136-1144.

Walker, J. E. (1992). "The NADH:ubiquinone oxidoreductase (complex I) of respiratory chains." Q Rev Biophys **25**(3): 253-324.

Watt, I. N., et al. (2010). "Bioenergetic cost of making an adenosine triphosphate molecule in animal mitochondria." Proc Natl Acad Sci U S A **107**(39): 16823-16827.

Wittig, I., et al. (2010). "Mass estimation of native proteins by blue native electrophoresis: principles and practical hints." Mol Cell Proteomics **9**(10): 2149-2161.

Xia, D., et al. (1997). "Crystal structure of the cytochrome bc1 complex from bovine heart mitochondria." Science **277**(5322): 60-66.

Yang, X. H. and B. L. Trumpower (1986). "Purification of a three-subunit ubiquinol-cytochrome c oxidoreductase complex from Paracoccus denitrificans." J Biol Chem **261**(26): 12282-12289.

Yankovskaya, V., et al. (2003). "Architecture of succinate dehydrogenase and reactive oxygen species generation." Science **299**(5607): 700-704.

Zhang, Z., et al. (1998). "Electron transfer by domain movement in cytochrome bc1." Nature **392**(6677): 677-684.

Zhu, J., et al. (2016). "Structure of mammalian respiratory complex I." Nature **536**(7616): 354-358.

Zickermann, V., et al. (2000). "The NADH oxidation domain of complex I: do bacterial and mitochondrial enzymes catalyze ferricyanide reduction similarly?" Biochim Biophys Acta **1459**(1): 61-68.

Zickermann, V., et al. (2015). "Structural biology. Mechanistic insight from the crystal structure of mitochondrial complex I." Science **347**(6217): 44-49.
